# Impaired tryptophan metabolism in the gastrointestinal tract of patients with critical coronavirus disease 2019

**DOI:** 10.3389/fmed.2022.941422

**Published:** 2022-08-10

**Authors:** Yoshihiro Yokoyama, Tomoko Ichiki, Tsukasa Yamakawa, Yoshihisa Tsuji, Koji Kuronuma, Satoshi Takahashi, Eichi Narimatsu, Hiroshi Nakase

**Affiliations:** ^1^Department of Gastroenterology and Hepatology, Sapporo Medical University School of Medicine, Sapporo, Japan; ^2^Department of General Medicine, Sapporo Medical University School of Medicine, Sapporo, Japan; ^3^Department of Respiratory Medicine and Allergology, Sapporo Medical University School of Medicine, Sapporo, Japan; ^4^Department of Infection Control and Laboratory Medicine, Sapporo Medical University School of Medicine, Sapporo, Japan; ^5^Department of Intensive Care Medicine, Sapporo Medical University School of Medicine, Sapporo, Japan

**Keywords:** COVID-19, gastrointestinal inflammation, fecal calprotectin, metabolome, microbiome, aryl hydrocarbon receptor, tryptophan, ACE2

## Abstract

**Introduction:**

Coronavirus disease 2019 (COVID-19) is still causing a global pandemic. But the mechanism of COVID-19 severity is not well elucidated.

**Materials and methods:**

We conducted two single-center observational studies of patients with COVID-19. In the first study, the enrolled patients were distinguished based on critical vs. non-critical COVID-19. We collected blood samples from the patients at admission to measure markers related to inflammation and thrombosis and stool samples to analyze the fecal microbiome, metabolome, and calprotectin level. In the second study, we collected ileum and colon tissue samples from patients with critical COVID-19 who required colonoscopy due to severe gastrointestinal symptoms and analyzed mucosal gene expression.

**Results:**

A total of 19 blood samples and 10 stool samples were collected. Interleukin (IL)-6 was the only serum inflammatory marker with significantly higher levels in the critical group than in the non-critical group. The fecal calprotectin level in the critical group was significantly higher than that in the non-critical group (*P* = 0.03), regardless of the presence of gastrointestinal symptoms. Stool metabolomic analysis showed that the level of indole-3-propionic acid, a ligand for aryl hydrocarbon receptor (AhR), was markedly decreased in the critical group compared to that in the non-critical group (*P* = 0.01). The expression of genes involved in tryptophan metabolism, including *ACE2*, *AHR*, *CARD9*, and *IL22*, was downregulated in the ileum of critical COVID-19 patients who required a colonoscopy.

**Discussion:**

Critical COVID-19 patients have gastrointestinal inflammation potentially caused by impaired tryptophan metabolism in the small intestine due to decreased expression of genes involved in tryptophan metabolism.

## Introduction

Coronavirus disease 2019 (COVID-19) was first reported in December 2019 and continues to cause a worldwide pandemic in 2022. COVID-19 patients often experience gastrointestinal symptoms, such as diarrhea, nausea and vomiting, anorexia, and abdominal pain (1). Our institution reported that abdominal pain is a risk factor for severe disease outcomes in COVID-19 (2). We have also reported a rare case of severe gastrointestinal mucosal damage during the treatment of COVID-19 pneumonia (3).

Severe acute respiratory syndrome coronavirus 2 (SARS-CoV-2) invades human cells expressing angiotensin-converting enzyme 2 (ACE2) (4). ACE2 is abundantly expressed in intestinal epithelial cells, such as the small and large intestines (4), and previous reports have shown that SARS-CoV-2 can infect the cells of the gastrointestinal tract (5–7). ACE2 plays an important role in the negative regulation of intestinal inflammation by controlling the absorption of tryptophan in the small intestine (8). Therefore, the presence of SARS-CoV-2 in the gastrointestinal tract could be involved in COVID-19 pathogenesis. Additionally, COVID-19 patients have persistent gut microbial dysbiosis, which correlates with severe COVID-19 outcomes (9, 10). Regarding the induction of inflammation, SARS-CoV-2 causes acute respiratory distress syndrome by activating the NLR family pyrin domain containing 3 (NLRP3) inflammasome, which activates interleukin (IL)-1β and IL-18 *via* caspase-1 (11, 12), and the NLRP3 inflammasome is known to be an important molecule involved in intestinal homeostasis (13). However, evidence relating to gastrointestinal damage in COVID-19 is still lacking, and the pathophysiology associated with this damage has not yet been clarified.

In the current study, we demonstrate that patients with critical COVID-19 have gastrointestinal inflammation and altered levels of fecal metabolites related to tryptophan metabolism. In accordance with this finding, we show that in the small intestine of critical COVID-19 patients, the expression levels of *ACE2* and genes that regulate tryptophan metabolism are decreased.

## Materials and methods

### Patients and sample collection for the first study

In the first study, we assessed adult patients hospitalized at Sapporo Medical University who were shown to be positive for SARS-CoV-2 infection by real-time PCR or antigen testing of nasopharynx or sputum samples. We divided the study population into a critical group and a non-critical group according to the NIH classification of COVID-19 severity (14). We obtained patient backgrounds and blood test findings from medical records.

For serological assays, the residual sera for routine blood tests were stored at −80°C. Stool samples were collected after informed consent was obtained and stored at 4°C for microbiome analysis and −80°C for metabolome analysis. A second stool sample was collected from those patients who were able to provide a sample at least 1 week after the first sample was collected.

### Serological assays

The levels of serum IL-18, TNF-α, ADAMTS13, VEGF, and ICAM-1 were determined using enzyme-linked immunosorbent assay (ELISA) kits (R&D Systems, MN, United States). The detailed methods are described in the Supplementary material. We also measured serum LRG levels, which is a biomarker in disease activity of inflammatory bowel disease, with a NANOPIA^®^ LRG Kit (Sekisui Medical, Tokyo, Japan). According to the product information, the reference value (mean ± 2 SD) for healthy subjects is 10.2 ± 3.72 μg/ml, and that for the active phase of patients with inflammatory bowel disease is > 16.0 μg/ml.

### Fecal calprotectin analysis

Fecal calprotectin (FCP) levels were measured using a Calprotectin ELISA Kit (Calprotectin Mochida). In brief, FCP levels were measured based on a sandwich enzyme immunoassay using a monoclonal antibody. FCP levels were calculated by measuring the absorbance with a spectrophotometer. The 95% confidence interval of FCP in healthy volunteers is 0–94 μg/g (mean value = 43.0 μg/g, SD = ± 26.0 μg/g), and the cutoff value for endoscopic remission in patients with ulcerative colitis is 240 μg/g (15). If the measured FCP value was under 30 μg/g, which is less than the sensitivity limit of the assay, the result was defined as 30 μg/g.

### Fecal microbiome analysis

For fecal microbiome analysis, 16S rRNA PCR amplification and gene sequencing were performed by professionals at Miyarisan Pharmaceutical Co., Ltd. (Tokyo, Japan). The 16S rRNA gene sequence data were processed using the Quantitative Insights Into Microbial Ecology 2 (QIIME 2 October 2019) pipeline (16). The detailed methods for the fecal microbiome analysis are provided in the Supplementary material.

### Fecal metabolome analysis

For metabolomic analysis, metabolites were analyzed by capillary electrophoresis time-of-flight mass spectrometry (CE-TOFMS) at Human Metabolome Technologies, Inc. (Yamagata, Japan). The detailed methods for the fecal metabolome analysis are provided in the Supplementary material.

### Patients and sample collection for the second study

In our second study, biopsy samples were obtained by colonoscopy from inflamed areas of the ileum or colorectum of critical COVID-19 patients in our hospital who required colonoscopy because of severe gastrointestinal symptoms. For the control group, we collected specimens of the normal ileum or colon mucosa of patients who underwent colorectal polypectomy.

### Mucosal gene expression analysis

We extracted messenger RNA from the ileal or colorectal biopsy samples, reverse transcribed it into complementary DNA, and analyzed gene expression by real-time PCR (see Supplementary material for more detailed descriptions). The gene expression relative to the control group was calculated using the ΔΔCT method and normalized to beta actin expression.

### Statistics

Normally distributed continuous variables were analyzed using unpaired Student’s *t*-test, and non-parametric data were analyzed using the Wilcoxon rank-sum test.

To analyze the diversity of the microbiome, one-way ANOVA with Tukey’s test was applied to compare the total number of genes in the extracted pathway by PICRUSt2 analysis. A Mann–Whitney U test was applied for α-diversity, and the Kruskal–Wallis test was applied for linear discriminant analysis (LDA) effect size (LEfSe). β-Diversity *p*-values were calculated using permutational analysis of variance (PERMANOVA). For metabolomic analysis, Welch’s *t*-test was applied for group comparison.

Two-sided *P*-values less than 0.05 were considered to indicate statistical significance. All analyses were performed using JMP version 15 (SAS Institute, Cary, NC, United States).

### Study approval

The current human study consisted of two single-center, prospective, observational studies. Both studies were conducted at Sapporo Medical University, Sapporo, Japan, and were approved by the ethical committee of Sapporo Medical University (IRB number: 312-75, 332-111). These studies were part of a comprehensive clinical trial that was registered in the University Hospital Medical Information Network Clinical Trial Registry (UMIN-CTR: 000041268, 000046106). All the patients provided written informed consent to participate in this study.

All the authors contributed to the collection and analysis of the data to ensure the accuracy and completeness of the data and fidelity to the protocol. These studies were conducted in accordance with the Declaration of Helsinki.

## Results

### Study patients

For the first study, we obtained informed consent from nineteen COVID-19 patients between July and November 2021. Of these patients, 13 were assigned to the non-critical group, and six patients were assigned to the critical group based on the National Institutes of Health (NIH) classification of COVID-19 severity (Table 1). Blood samples were collected from all 19 patients. Stool samples were collected from 10 of the 19 patients (*n* = 5 in the critical group and *n* = 5 in the non-critical group). The clinical characteristics of these 10 patients are shown in Supplementary Table 1.

**TABLE 1:** Clinical characteristics of the COVID-19 patients included in the study.

	All (*n* = 19)	Non-critical *(n* = 13)	Critical (*n* = 6)
**General characteristics**			
Age, y	49.6 ± 10.74	46.8 ± 11.49	55.5 ± 6.06
Male, %	13 (68.4)	8 (61.5)	5 (83.3)
BMI	27.34 ± 4.59	27.1 ± 5.22	27.8 ± 3.18
Current smoking, %	3 (15.8)	2 (15.4)	1 (16.7)
**COVID-19 disease severity category**			
Mild, %	1 (5.3)	1 (7.7)	
Moderate, %	8 (42.1)	8 (61.5)	
Severe, %	4 (21.1)	4 (30.8)	
Critical, %	6 (28.6)		6 (100)
**Comorbidity**			
Hypertension, %	8 (42.1)	4 (30.8)	4 (66.7)
Diabetes mellitus, %	2 (10.5)	0 (0)	2 (33.3)
Hyperlipidemia, %	4 (21.1)	2 (15.4)	2 (33.3)
COPD, %	1 (5.3)	1 (7.7)	0 (0)
Ischemic heart disease, %	1 (5.3)	1 (7.7)	0 (0)
Ulcerative colitis, %	1 (5.3)	1 (7.7)	0 (0)
**Symptoms at hospitalization**			
High fever > 37.5°C, %	19 (100)	13 (100)	6 (100)
General fatigue, %	12 (63.2)	9 (69)	3 (50)
Cough, %	12 (63.2)	9 (69)	3 (50)
Diarrhea, %	5 (26.3)	4 (30.7)	1 (16.7)
Appetite loss, %	4 (21.1)	3 (23.1)	1 (16.7)
Abdominal pain, %	2 (10.6)	1 (7.7)	1 (16.7)
Nausea, %	2 (10.6)	2 (15.4)	0 (0)
**Therapeutics after admission**			
Steroids, %	15 (78.9)	9 (69.2)	6 (100)
Antiviral antibodies, %	11 (57.9)	6 (46.2)	5 (83.3)
Anti-inflammatory drugs, %	11 (57.9)	6 (46.2)	5 (83.3)
Anti-SARS-CoV-2 antibody cocktail, %	3 (15.8)	3 (23.1)	0 (0)

Data are expressed as the mean ± SD for normally distributed data or the median (interquartile range) for non-normally distributed data. Percentages may not add up to 100% due to rounding or overlap. BMI, body mass index; COPD, chronic obstructive pulmonary disease.

For the second study, biopsy samples were collected from a total of three patients with COVID-19 and severe gastrointestinal symptoms who required colonoscopy between June 2020 and November 2021. In total, 2 individuals had both inflamed ileal and rectum mucosa collected and 1 individual had only an inflamed rectum mucosa collected. We summarize the characteristics of the three patients who required colonoscopy in Supplementary Table 2. As a healthy control group, three tissue specimens of the ileum and five tissue specimens of the colon were collected from patients undergoing polypectomy.

### Serum biomarkers

First, the blood test results at admission are shown in Supplementary Table 3. The critical COVID-19 group had lower total protein, albumin, calcium, and lymphocyte levels and higher lactic dehydrogenase, blood urea nitrogen, and d-dimer levels than the non-critical group. Next, we further measured the levels of the serum biomarkers IL-18 and tumor necrosis factor (TNF)-α as proinflammatory cytokines related to the NLRP3 inflammasome, and the levels of vascular endothelial growth factor (VEGF), intercellular adhesion molecule (ICAM)-1, and a disintegrin and metalloproteinase with thrombospondin type 1 motif member 13 (ADAMTS13) as the serum biomarkers of endothelial inflammation and microvascular thrombosis. Supplementary Table 4 shows the serum levels of these biomarkers in the healthy controls and COVID-19 patients. Compared to the healthy controls, the COVID-19 patients had significantly higher IL-18, TNF-α, and VEGF levels, suggesting that SARS-CoV-2 infection may activate NLRP3 inflammasome-related inflammation. However, when comparing the critical and non-critical COVID-19 groups, there was no significant difference in serum IL-18, TNF-α, or VEGF levels, and only serum IL-6 levels were significantly elevated in the critical group compared to the non-critical group (Table 2). Serum leucine-rich α-2-glycoprotein (LRG) levels were elevated in our COVID-19 patients (22.7 ± 12.2 μg/ml). However, there was no significant difference in the LRG level between the critical and non-critical groups.

**TABLE 2:** Levels of serum biomarkers associated with inflammation and thrombosis in critical and non-critical COVID-19 patients.

	All (*n* = 19)	Non-critical (*n* = 13)	Critical (*n* = 6)	*P*-value
Duration from infection to sample collection (days)	7 (5–11)	8 (6–12.5)	13 (11–13.3)	0.09
IL-18, pg/ml	402.0 ± 133.52	399.8 ± 133.4	406.8 ± 146.3	0.92
TNF-α, pg/ml	9.37 (7.93, 11.53)	9.4 (7.57, 13.16)	9.2 (8.29, 12.98)	0.76
VEGF, pg/ml	402.9 (266.7, 787.0)	382.1 (223.7, 519.3)	1133.7 (378.2, 1678.3)	0.11
**IL-6, pg/ml**	**12.4 (4.96, 77.15)**	**10.5 (4.36, 19.05)**	**93.2 (12.6, 2285)**	**0.03**
ADAMTS13, ng/ml	749.7 ± 135.88	762.1 ± 139.2	722.7 ± 136.7	0.57
ICAM-1, ng/ml	224.3 ± 84.3	202.9 ± 76.92	270.6 ± 87.36	0.11
LRG, μg/ml	22.7 ± 12.2	22.9 ± 12.6	22.1 ± 12.4	0.90

Data are expressed as the mean ± SD for normally distributed data or the median (interquartile range) for non-normally distributed data. Statistical analysis was performed by using unpaired Student t-test and Wilcoxon rank-sum test. Non-critical and Critical groups were included in this comparison. Statistical significance was accepted as P < 0.05. Data in bold are statistically significant. IL, interleukin; TNF, tumor necrosis factor; VEGF, vascular endothelial growth factor; ADAMTS13, a disintegrin, and metalloproteinase with thrombospondin type 1 motif member 13; ICAM-1, intercellular adhesion molecule-1; LRG, leucine-rich α-2-glycoprotein.

### Fecal calprotectin levels

**FIGURE 1:**
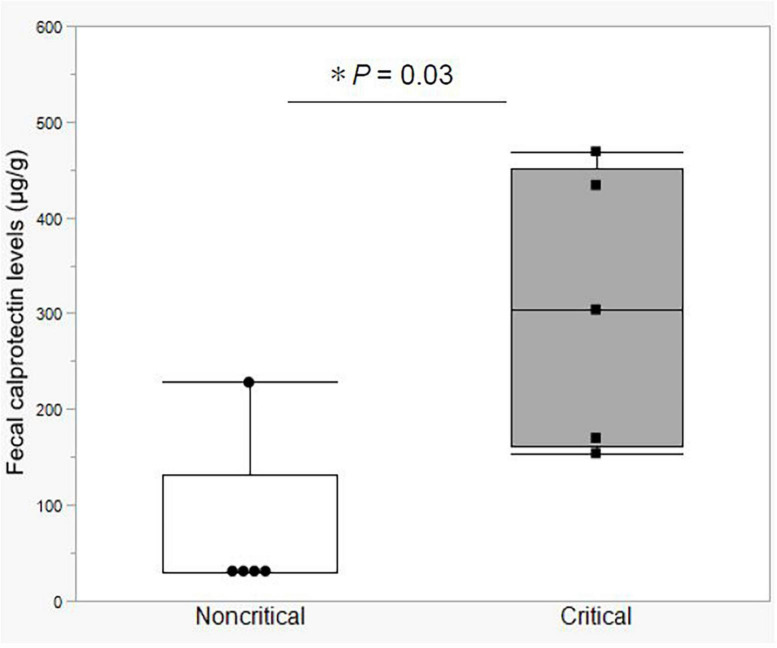
Fecal calprotectin levels between the non-critical and critical COVID-19 groups. Comparison of fecal calprotectin levels between the non-critical (*n* = 5) and critical (*n* = 5) COVID-19 groups. Box-and-whisker plots show the median, 25th and 75th percentiles, with whiskers extending to the minimum and maximum values. Each dot indicates individual values. Statistical analysis was performed using Wilcoxon rank-sum test. Statistical significance was accepted as *P* < 0.05. If the measured FCP value was under 30 μg/g, which is less than the sensitivity limit of the assay, the result was defined as 30 μg/g.

In the first study, we compared fecal calprotectin (FCP) levels in the stool of critical and non-critical COVID-19 patients. Notably, the FCP level was elevated in all the critical COVID-19 patients (mean value = 306.2 μg/g, SD = ± 145.4 μg/g). The mean FCP level in the critical group was significantly higher than that in the non-critical group, which included a patient with ulcerative colitis in clinical remission and an FCP level of 248 μg/g (Figure 1). These data indicated that the critical COVID-19 patients had gastrointestinal tract inflammation.

### Fecal microbiome analysis

**FIGURE 2:**
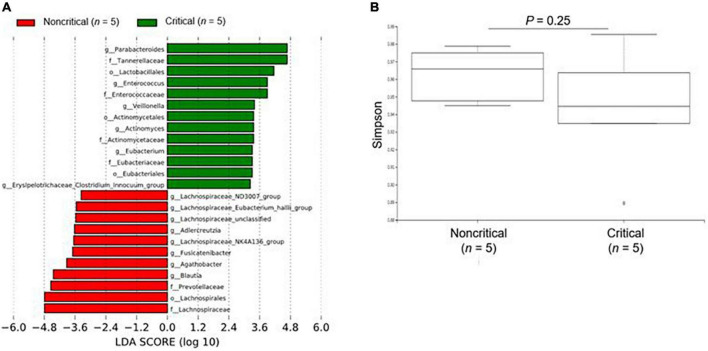
Microbiome analysis of fecal contents from COVID-19 patients. **(A)** The enriched gut microbiota constituents were identified by linear discriminant analysis (LDA) effect size (LEfSe). The histogram shows taxa with LDA scores with (log 10) values > 3 and *P* < 0.05. g_ means genus, f_ means family, and o_ means order. **(B)** The α-diversity represented by the Simpson index. The vertical axis shows the Simpson index. Box-and-whisker plots show the median, 25th and 75th percentiles, with whiskers extending to the minimum and maximum values. Data were analyzed using Mann–Whitney *U* test. Statistical significance was accepted as *P* < 0.05.

We show the results of the microbiome analysis in Figure 2 and Supplementary Figure 1. For the microbiome analysis, LEfSe revealed that intestinal bacteria in the non-critical and critical groups were significantly differentially abundant (Figure 2A). In the critical group, *Parabacteroides*, *Tannerellaceae*, and *Lactobacillales* were the most enriched compared to the non-critical group. There was a slightly lower microbial α diversity in the critical group than in the non-critical group, but the difference was not significant (*P* = 0.25, Figure 2B).

### Fecal metabolome analysis

**FIGURE 3:**
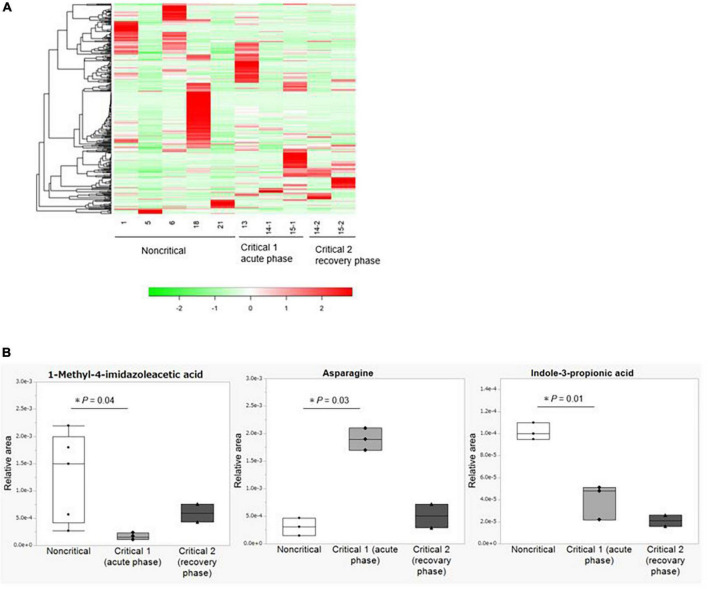
Metabolome analysis of fecal contents from COVID-19 patients. **(A)** Hierarchical cluster analysis (HCA) of the metabolome data. From left to right: the non-critical group (*n* = 5), the critical group in the acute phase (critical 1, *n* = 3), and the critical group in the recovery phase (critical 2, *n* = 2). **(B)** Fecal metabolites with significant differences between the non-critical and critical groups. From left to right: the non-critical group (*n* = 5), the critical group in the acute phase (critical 1, *n* = 3), and the critical group in the recovery phase (critical 2, *n* = 2). Asparagine and Indole-3-propionic acid in the non-critical group were not detected in two of the five cases. Statistical analysis was performed by using Welch’s *t*-test. Statistical significance was accepted as *P* < 0.05.

We show the results of the microbiome analysis in Figure 3 and Supplementary Figure 2. In the metabolome analysis, we performed two comparisons: first, we compared samples collected from the five non-critical COVID-19 patients with those collected from three critical COVID-19 patients in the acute phase (critical 1), which we defined as the period when the patients required ventilation or extracorporeal membrane oxygenation. Second, we compared samples collected from two critical COVID-19 patients during the acute phase with those collected from the same patients in the recovery phase (critical 2), which we defined as the period when the patients were weaned from these treatments. The duration from infection to sample collection in the acute phase is shown in Supplementary Figure 1, while the duration from infection to sample collection in the recovery phase was 21 and 24 days, respectively. We analyzed the differential abundance of all 310 identified fecal metabolites among the groups (Supplementary Table 5). There were no differences among the groups based on heatmap hierarchical cluster analysis (HCA) of the metabolomic data (Figure 3A). Principal component analysis (PCA) of the metabolomic data also showed no significant changes in fecal metabolite levels among the three groups (Supplementary Figure 2). Three out of 310 identified fecal metabolites showed significantly differential abundances (*P* < 0.05) between the non-critical and critical groups in the acute phase (Figure 3B). Among them, indole-3-propionic acid was markedly depleted in the critical group in the acute phase (*P* = 0.01), and this trend was persistent during the recovery phase. Other metabolites involved in tryptophan metabolisms, such as tryptophan, tryptamine, and indole-3-acetic acid, were not significantly different between the non-critical and critical groups (shown in Supplementary Table 5).

### Mucosal gene expression

**FIGURE 4:**
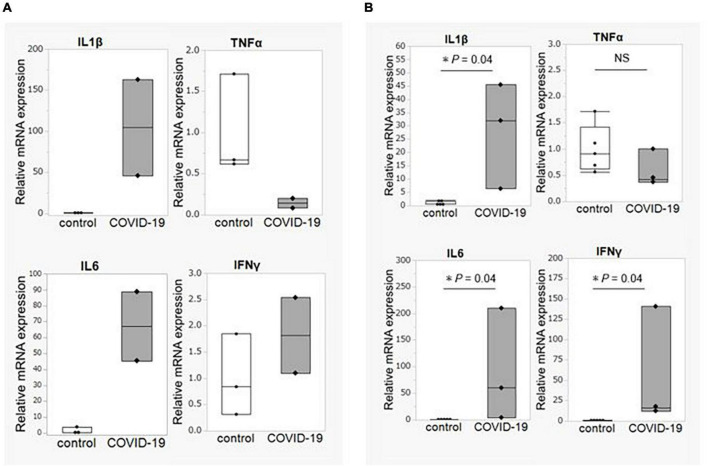
Intestinal mucosal gene expression of inflammatory cytokines. **(A)** Comparison of gene expression of inflammatory cytokines between COVID-19 patients (*n* = 2) and healthy controls (*n* = 3) in the ileum. **(B)** Comparison of gene expression of inflammatory cytokines between COVID-19 patients (*n* = 3) and healthy controls (*n* = 5) in the colon. Statistical analysis was performed using the Wilcoxon rank sum-test. Statistical significance was accepted as *P* < 0.05. NS means *P* > 0.05. IL, interleukin; TNF, tumor necrosis factor; IFN, interferon.

We obtained biopsy tissue from three critical COVID-19 patients who required a colonoscopy (ileum = 2, colon = 3) and from healthy control participants (ileum = 3, colon = 5). First, the expression of inflammatory cytokine genes was compared. The expression levels of *IL1B* and *IL6* were significantly elevated in both the ileum and the colon, whereas the *TNFA* expression level was not elevated in the patient group compared to that in the control group (Figures 4A,B).

**FIGURE 5:**
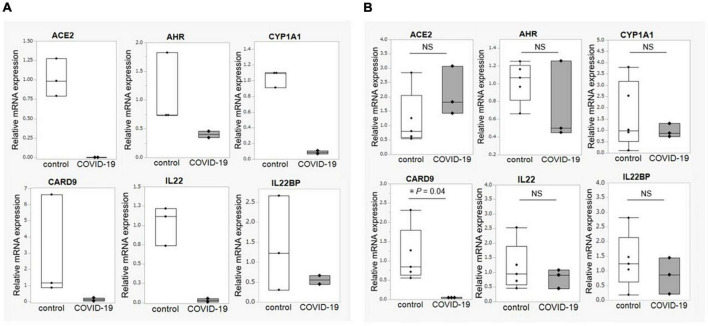
Intestinal mucosal gene expression associated with tryptophan metabolism. **(A)** Comparison of gene expression associated with tryptophan metabolism between COVID-19 patients (*n* = 2) and healthy controls (*n* = 3) in the ileum. **(B)** Comparison of gene expression associated with tryptophan metabolism between COVID-19 patients (*n* = 3) and healthy controls (*n* = 5) in the colon. Statistical analysis was performed using the Wilcoxon rank sum-test. Statistical significance was accepted as *P* < 0.05. NS means *P* > 0.05. ACE2, angiotensin-converting enzyme-2; AHR, aryl hydrocarbon receptor; CYP1A1, Cytochrome P450 1A1; CARD9, caspase recruitment domain-containing protein 9.

Next, based on the metabolomic analysis showing lower levels of indole-3-propionic acid in the critical COVID-19 group and the fact that indole-3-propionic acid is a metabolite of tryptophan, we analyzed the expression of genes involved in tryptophan metabolism. The expression of *ACE2*, *AHR*, and *CARD9* was decreased in the ileum of the COVID-19 patients, and the expression of *IL22*, a cytokine regulated by aryl hydrocarbon receptor (AhR), was also decreased in the ileum of the COVID-19 patients (Figures 5A,B). We further examined the gene expression of IL22 binding protein (*IL22BP*), an antagonist of IL-22. There was no difference in the expression of *IL22BP* between the control and COVID-19 groups in either the ileum or colon. These results provide more supporting data that the deficiency in the AhR pathway, rather than IL-22BP, as the cause of decreased *IL22* expression in COVID-19 patients.

## Discussion

This study showed that gastrointestinal inflammation occurs in patients with critical COVID-19. Even in the absence of abdominal symptoms, FCP may be a predictive biomarker of infection severity. In addition, the mechanism of gastrointestinal damage in patients with COVID-19 may involve impaired tryptophan metabolism due to the decreased expression of *ACE2*, *AHR*, and *CARD9* in the small intestinal mucosa.

COVID-19 is a highly lethal disease that is currently causing a worldwide pandemic, with an in-hospital mortality rate of approximately 15–20% (17). Therefore, predicting the severity of COVID-19 is an important clinical issue. Severe COVID-19 predictors include elevated serum C-reactive protein, lactate dehydrogenase, and D-dimer (18). Moreover, severe COVID-19 patients requiring intensive care in the hospital have high plasma IL-2, IL-7, IL-10, and TNF-α levels (19). In addition to these reports, our data suggest that FCP content may be a biomarker of COVID-19 severity. Notably, FCP can be easily measured in routine clinical practice.

The microbiome analysis showed clear differences in the intestinal microbiota composition between the critical and non-critical groups. Yeoh et al. examined the gut microbiota of COVID-19 patients and reported that gut bacteria constituents with known immunomodulatory potential, such as *Faecalibacterium prausnitzii, Eubacterium rectale*, and several bifidobacterial species were depleted (10). In contrast, our data showed that protective microbes of the gastrointestinal tract, such as *Lactobacillales*, were enriched in the critical group compared to the non-critical group. These data suggest that gut microbiota constituents shift to an anti-inflammatory community structure to counteract the proinflammatory environment in patients with critical COVID-19. We considered that the increase in gut microbiota with anti-inflammatory capacity in critical COVID-19 patients, even to the point of reduced alpha diversity resulting from impaired tryptophan metabolism, suggests a protective response *in vivo*.

Our metabolomic analysis and mucosal gene expression data provide critical insight into the mechanism of COVID-19-induced gastrointestinal damage. First, our mucosal PCR results showed decreased gene expression of ACE2 in the ileum. ACE2 regulates tryptophan absorption in the small intestine and modulates the intestinal microbiota by enhancing the expression of antimicrobial peptides (8). The observed reduction in ACE2 expression by SARS-CoV-2 infection could lead to Paneth cell dysfunction, which is associated with decreased production of antimicrobial peptides and inflammation. Of note, our metabolomic analysis indicated a decrease in indole-3-propionic acid content in the critical COVID-19 patients, while other tryptophan metabolites were not clearly different between non-critical and critical COVID-19 patients. The mucosal gene expression analysis showed both AHR and the downstream IL-22 gene were decreased in critical COVID-19 group. Taken together, these data indicated that decreased *AHR* expression can further reduce ACE2 expression and accelerate tryptophan metabolism impairment (20). We also found decreased gene expression of cytochrome P450 (CYP) 1A1, an indicator of AhR activity (20), and caspase recruitment domain-containing protein 9 (CARD9), a gene that regulates the conversion of tryptophan to an AhR ligand (21). These results suggested that the decreased ACE2 expression and impaired AhR pathway in the small intestine were responsible for inflammation in the gastrointestinal tracts of the COVID-19 patients (Supplementary Figure 3). We considered that the mucosal damage to the small intestine by SARS-CoV-2 contributes to the impaired tryptophan metabolism that occurs in critical COVID-19 patients, and this impairment of tryptophan metabolism could further lead to a breakdown in the maintenance of intestinal immune mechanisms. In mucosal gene expression analysis, the ACE2 and AhR pathways were downregulated in the ileum, but not in the colon. These results suggest that the small intestine is abundant in ACE2 and more susceptible to SARS-CoV-2 infection than the colon and that the AhR pathway is characteristic of the small intestine. Thus, the small intestine is the most important area of gastrointestinal mucosal damage in COVID-19 patients.

As another aspect of gastrointestinal damage in COVID-19 patients, there have been a few reports of ischemic enteritis-like symptoms (22). In addition, immunothrombosis derived from vascular endothelial damage is known to be associated with multiple organ failure and severity in COVID-19 patients (23). In this study, serum biomarkers of vascular endothelial damage were not significantly differentially expressed between the critical and non-critical groups, but we found elevated levels of IL-6 in the serum and increased expression of IL-6 and IL-1β in the intestinal mucosa of patients with critical COVID-19. IL-6 and IL-1β promote thrombus formation in the vascular endothelium (24, 25). Furthermore, both IL-6 and IL-1β have been reported to be involved in thrombosis in SARS-CoV-2 infection (26). Therefore, these cytokines may also contribute to local microcirculation disturbance and thrombus formation related to gastrointestinal damage.

Based on our data, there are two possible clinical implications. First, tryptophan supplementation or improvement of the tryptophan metabolic pathway may prevent severe COVID-19; second, blockade of IL-6 and IL-1β may prevent severe COVID-19 by protecting against circulatory disturbance caused by thrombosis. To resolve these issues, further basic and clinical studies are required.

The limitation of this study is its small sample size. The prevalence of COVID-19 has been fluctuating throughout the pandemic, and the pandemic in Japan has had a relatively low impact compared to that in other countries. Furthermore, it is recommended that colonoscopy should be avoided as much as possible in COVID-19 patients to prevent the spread of infection (27), which made it even more difficult to obtain biopsy tissue specimens.

In conclusion, patients with critical COVID-19 have gastrointestinal inflammation potentially caused by impaired tryptophan metabolism due to decreased ACE2 expression and AhR pathway disruption.

## Data availability statement

The datasets presented in this study can be found in online repositories. The names of the repository/repositories and accession number(s) can be found below: https://www.ncbi.nlm.nih.gov/, PRJDB13936.

## Ethics statement

The studies involving human participants were reviewed and approved by the Sapporo Medical University. The patients/participants provided their written informed consent to participate in this study.

## Author contributions

YY and HN: study concept and design. YY, TY, and TI: acquisition of data. YY and TI: analysis and interpretation of data. YY: drafting of the manuscript. TI, YT, KK, ST, and EN: critical revision of the manuscript. HN: study supervision. All authors contributed to the article and approved the submitted version.
