# Supplementary material for: Impaired tryptophan metabolism in the gastrointestinal tract of patients with critical coronavirus disease 2019
Source: Front Med (Lausanne). 2022 Aug 10;9:941422. doi: 10.3389/fmed.2022.941422 (PMC9399458; doi:10.3389/fmed.2022.941422)
Supplement: Supplementary file 1 [file Data_Sheet_1.pdf]

# *Supplementary Material*

## 1. Supplementary Materials and Methods

### Serum assays

We performed serum assays of interleukin (IL)-18, a disintegrin and metalloproteinase with thrombospondin type 1 motif member 13 (ADAMTS13), vascular endothelial growth factor (VEGF), tumor necrosis factor (TNF)-α, and intercellular adhesion molecule (ICAM)-1 using enzyme-linked immunosorbent assay (ELISA) kits for human serum and plasma (Quantikine, R&D Systems, MN, USA). Samples and standards of recombinant human IL-18, ADAMTS13, VEGF, TNF-α, or ICAM-1 protein were added to microtiter plate wells precoated with IL-18, ADAMTS13, VEGF, TNF-α, or ICAM-1 antibody and incubated for two hours at room temperature. Each well was washed and then incubated with IL-18, ADAMTS13, VEGF, TNF-α, or ICAM-1 conjugate for two hours at room temperature. Wells were washed, and substrate solution was added. After incubation for 20-30 min at room temperature according to each assay instruction, the enzyme reactions were stopped, and then the optical density of each well was determined with a microplate reader set to 450 nm. The data were subtracted by the readings at 540 nm. IL-18, ADAMTS13, VEGF, TNF-α, and ICAM-1 concentrations were determined by comparison to the standard curve. The intra-assay coefficients of variation for IL-18, ADAMTS13, VEGF, TNF-α, and ICAM-1 were 6.72%, 7.8%, 13.9%, 4.51%, and 3.31%, respectively.

**Fecal microbiome analysis**

Genomic DNA from fecal samples was extracted using NucleoSpin® DNA Stool (Macherey-Nagel, Duren, Germany) according to the manufacturer's instructions and quantified using a Qubit 4 Fluorometer (Promega, Madison, WI, USA). 16S rRNA gene sequencing PCR and data processing were based on a previously described method (1). Taxonomy classification for the microbiome was calculated by comparing all amplicon sequence variants with SILVA 138.1 using the q2-feature-classifier (2). α-diversity and β-diversity were calculated using QIIME2 (3). Principal coordinate analysis (PCoA) was applied to the distance matrices to create a two-dimensional plot. We used the linear discriminant analysis (LDA) effect size approach to identify bacterial taxa that differed significantly between the critical group and noncritical group (4). Taxa groups with > 3 log 10 LDA scores were considered significantly enriched at a $P$ value $< 0.05$.

**Fecal metabolome analysis**

Fecal metabolome analysis was performed by Human Metabolome Technologies, Inc. (HMT). Approximately 30-50 mg of feces were mixed with 500 µL of Milli-Q water containing internal standards (H3304-1002, HMT, Tsuruoka, Yamagata, Japan). The mixture was centrifuged at 2,300 ×g and 4 °C for five min, after which 80 µL of the supernatant was centrifugally filtered through a Millipore 5-kDa cutoff filter (ULTRAFREE MC PLHCC, HMT) at 9,100 ×g and 4 °C for 120 min to remove macromolecules. Subsequently, the filtrate was evaporated to dryness under vacuum and reconstituted in 20 µL of Milli-Q water for metabolome analysis. Metabolome analysis was conducted according to HMT's Basic Scan package using capillary electrophoresis time-of-flight mass spectrometry (CE-TOFMS) based on methods

described previously (5,6). Briefly, CE-TOFMS analysis was carried out using an Agilent CE capillary electrophoresis system equipped with an Agilent 6210 time-of-flight mass spectrometer (Agilent Technologies, Inc., Santa Clara, CA, USA). The systems were controlled by Agilent G2201AA ChemStation software version B.03.01 (Agilent Technologies) and connected by a fused silica capillary (50 μm i.d. × 80 cm total length) with commercial electrophoresis buffer (H3301-1001 and I3302-1023 for cation and anion analyses, respectively, HMT) as the electrolyte. The spectrometer was scanned from m/z 50 to 1,000, and peaks were extracted using MasterHands automatic integration software (Keio University, Tsuruoka, Yamagata, Japan) to obtain peak information, including m/z, peak area, and migration time (7). Signal peaks corresponding to isotopomers, adduct ions, and other product ions of known metabolites were excluded, and the remaining peaks were annotated according to HMT's metabolite database based on their m/z values and migration times. Areas of the annotated peaks were then normalized to internal standards and sample amount to obtain relative levels of each metabolite. Hierarchical cluster analysis (HCA) and principal component analysis (PCA) (8) were performed by HMT's proprietary MATLAB and R programs, respectively.

**Mucosal gene expression analysis**

All samples obtained from the patients were quickly put into RNAprotect Tissue Reagent (QIAGEN, Hilden, Germany) after biopsy and stored at -80 °C until analysis.

Total RNA was extracted from the biopsy tissues using TRIzol reagents (Invitrogen, Carlsbad, CA, USA), and RNA concentrations were measured at 260/280 nm using UV

spectrophotometry. RNA from each sample was reverse transcribed using SuperScript IV VILO Master Mix (Invitrogen, Carlsbad, CA, USA). Synthesized complementary DNA equivalent to 1000 ng of RNA was mixed with Applied Biosystems™ PowerUp™ SYBR™ Green Master Mix (Applied Biosystems, Foster City, CA, USA) in wells up to a volume of 20 μl. The reaction conditions used for quantitative real-time PCR were as follows: 95 °C for 10 min followed by 40 cycles of 95 °C for 15 s and 60 °C for 60 s. The primers used for real-time PCR are shown in Table S6.

## 2. Supplementary Figures

## Supplementary Figure 1

**A**

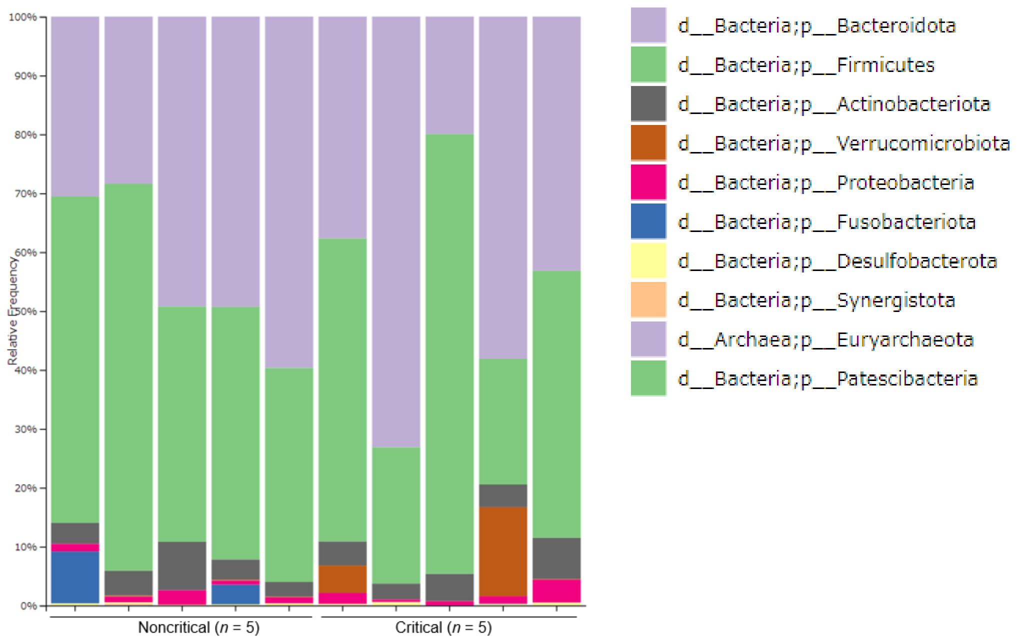


**B**

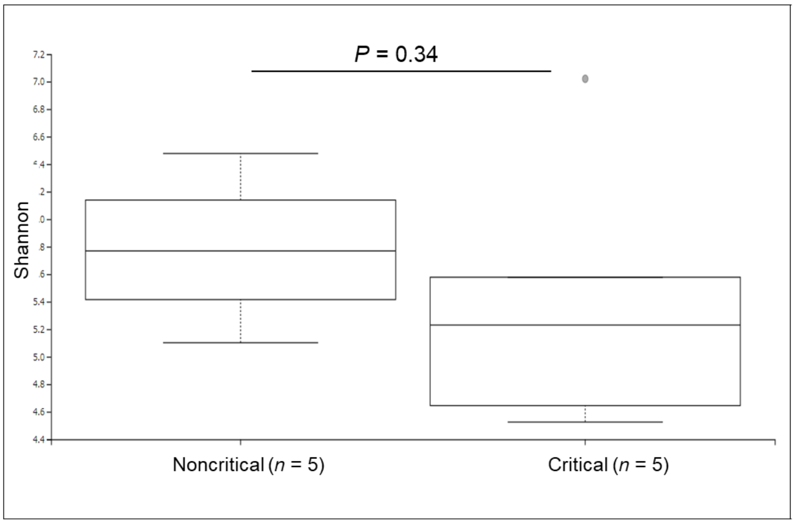

**C**

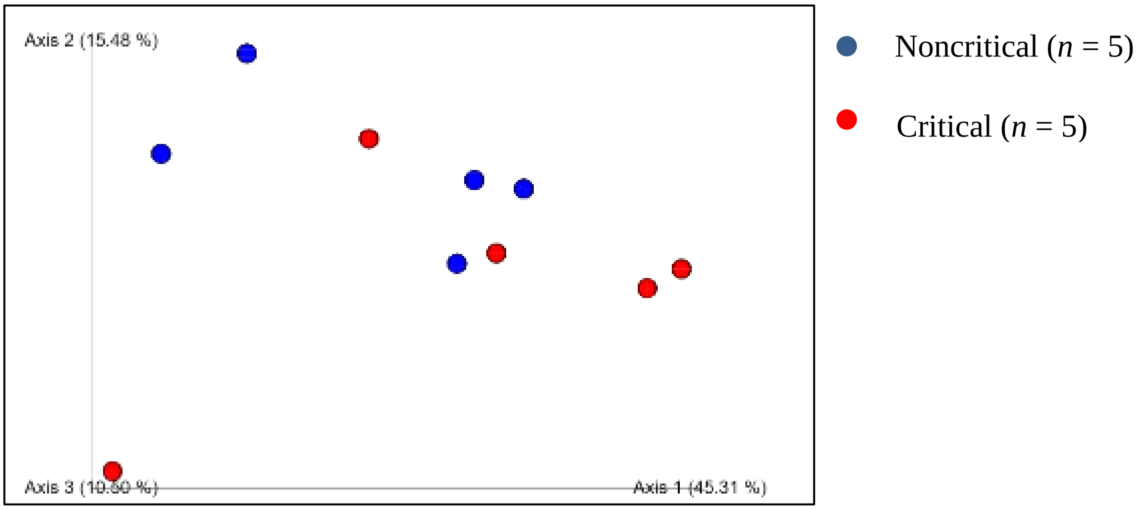


**(A)** Gut microbiota at the phylum level in two groups of COVID-19 patients. p_ means phylum.

**(B)** α-diversity represented by the Shannon index. The vertical axis shows the Shannon index. Box-and-whisker plots show the median, 25$^{th}$, and 75th percentiles, with whiskers extending to the minimum and maximum values. Data were analyzed using the Mann-Whitney U test. Statistical significance was accepted as $P < 0.05$. The Shannon index was slightly lower in the critical group than in the noncritical group, but the difference was not statistically significant ($P = 0.34$).

**(C)** Principal coordinates analysis (PCoA) derived from unweighted UniFrac distances. Statistical analysis was performed by using permutational analysis of variance (PERMANOVA). Statistical significance was accepted as $P < 0.05$. Comparison between the noncritical and critical groups showed no significant difference ($P =$ 0.28).

**Supplementary Figure 2**

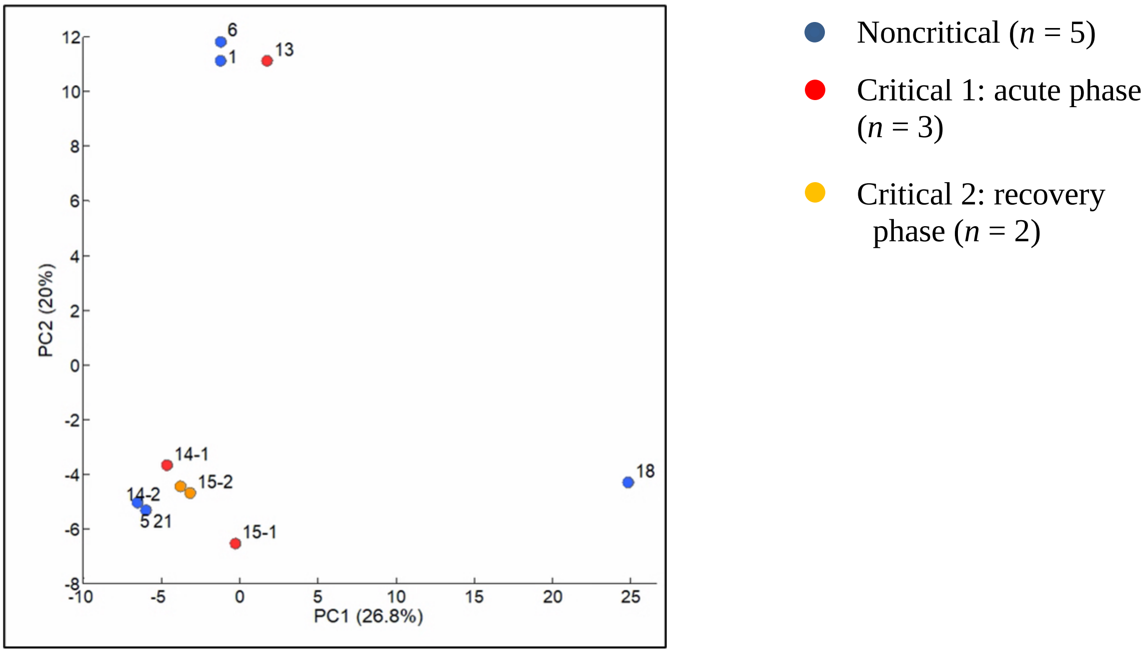


Principal component analysis (PCA) of metabolomic data from noncritical and critical COVID-19 patients. There were no significant changes in fecal metabolite levels among the three groups.

**Supplementary Figure 3**

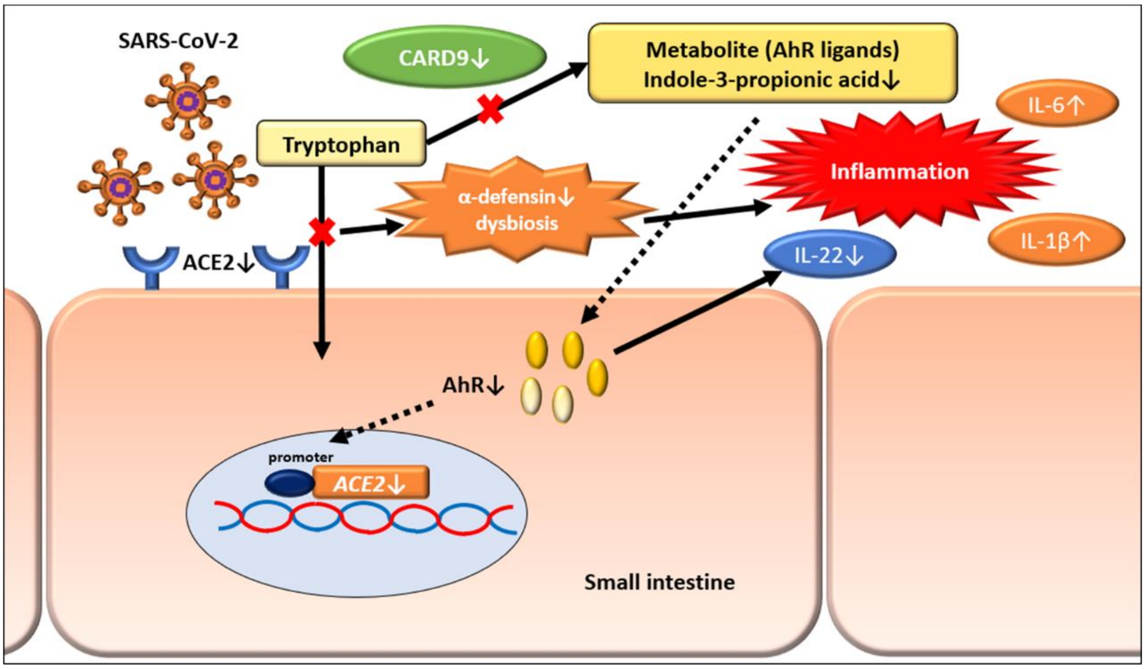


Possible mechanisms of gastrointestinal inflammation in patients with critical COVID-19.

## 3. Supplementary Tables

**Supplementary Table 1. Characteristics of 10 patients with COVID-19 from whom stool samples were obtained**

| | **All (*n* = 10)** | **Noncritical (*n* = 5)** | **Critical (*n* = 5)** |
|---|---|---|---|
| **GENERAL CHARACTERISTICS** | | | |
| Age, yo | 50.9 ±11.1 | 44.4 ± 12.3 | 57.4 ± 4.34 |
| Male, % | 6 (60) | 2 (40) | 4 (80) |
| BMI | 28.5 ±5.38 | 28.8 ± 7.34 | 28.3 ± 3.34 |
| Current smoking, % | 2 (20) | 1 (20) | 1 (20) |
| **DISEASE SEVERITY CATEGORY** | | | |
| Mild, % | 0 (0) | | |
| Moderate, % | 3 (30) | 3 (60) | |
| Severe, % | 2 (20) | 2 (40) | |
| Critical, % | 5 (50) | | 6 (100) |
| **DURATION FROM INFECTION TO SAMPLE COLLECTION** | | | |
| Days | 10.5 (5.5-12.8) | 8 (5-11) | 12 (10.5-18) |
| **COMORBIDITY** | | | |
| Hypertension, % | 4 (40) | 1 (20) | 3 (60) |
| Diabetes Mellitus, % | 2 (20) | 0 (0) | 2 (40) |

| | | | |
|---|---|---|---|
| Hyperlipidemia, % | 3 (30) | 1 (20) | 2 (40) |
| COPD, % | 1 (10) | 1 (20) | 0 (0) |
| Ischemic Heart Disease, % | 1 (10) | 1 (20) | 0 (0) |
| Ulcerative Colitis, % | 1 (10) | 1 (20) | 0 (0) |
| **SYMPTOMS AT HOSPITALIZATION** | | | |
| High Fever >37.5 °C, % | 10 (100) | 5 (100) | 5 (100) |
| General Fatigue, % | 6 (60) | 4 (80) | 2 (40) |
| Cough, % | 4 (40) | 2 (40) | 2 (40) |
| Diarrhea, % | 1 (10) | 1 (20) | 0 (0) |
| Appetite Loss, % | 1 (10) | 1 (20) | 0 (0) |
| Abdominal Pain, % | 1 (10) | 0 (0) | 1 (20) |
| Nausea, % | 2 (10) | 2 (40) | 0 (0) |
| **THERAPEUTICS AFTER ADMISSION** | | | |
| Steroids, % | 10 (100) | 5 (100) | 5 (100) |
| Anti-virus Antibodies, % | 6 (60) | 2 (40) | 4 (80) |
| Anti-inflammatory Drugs, % | 7 (70) | 3 (60) | 4 (80) |
| Anti-SARS-CoV-2 Ab Cocktail, % | 1 (10) | 1 (20) | 0 (0) |
| Data are expressed as the mean ± SD for normally distributed data or median (IQR) for nonnormally distributed data. | | | |

**Supplementary Table 2. Characteristics of three patients from whom mucosal biopsies were obtained**

| No | Age | Gender | BMI | Comorbidity* | GI symptoms | Duration from infection to the onset of GI symptoms (days) | Duration from infection to biopsy (days) | Biopsy site | Severity | Prognosis |
|---|---|---|---|---|---|---|---|---|---|---|
| 1 | 68 | Male | 26.1 | HT, DM | watery diarrhea | 25 | 33 | rectum | critical | dead |
| 2* | 63 | Male | 33.7 | HT, DM, asthma | watery diarrhea | 30 | 38 | ileum/rectum | critical | dead |
| 3 | 71 | Male | 19.9 | Prostate cancer | watery diarrhea | 16 | 19 | ileum/rectum | critical | dead |

Abbreviations; HT: Hypertension, DM: Diabetes mellitus, GI: Gastrointestinal tract.

*****Number 2 is a case that has already been published (3).

| **Supplementary Table 3. Laboratory data on admission** | | | | |
|---|---|---|---|---|
| | **All (*n* = 19)** | **Noncritical (*n* = 13)** | **Critical (*n* = 6)** | ***P* value** |
| White blood cells,/uL | 5000 (4100, 11900) | 4800 (4100, 9100) | 11250 (5125, 14075) | 0.19 |
| Neutrocytes,/uL | 3619 (2513, 10282) | 3395 (2464.5, 7227) | 9821 (4272, 12683.5) | 0.13 |
| **Lymphocytes,/uL** | **869 (666, 1160)** | **949 (748.5, 1372.5)** | **663 (517.8, 764)** | **0.01** |
| Hemoglobin, g/dL | 14.6 ± 1.73 | 14.9 ± 1.67 | 13.9 ± 1.84 | 0.28 |
| Platelets, x10$^4$/uL | 22.0 ± 9.30 | 20 ± 8.44 | 26.5 ± 10.3 | 0.16 |
| **Total Protein, g/dL** | **6.41 ± 0.81** | **7.7 ± 0.65** | **5.77 ± 0.80** | **0.01** |
| **Albumin, g/dL** | **3.28 ± 0.74** | **3.83 ± 0.51** | **2.65 ± 0.13** | **<0.001** |
| Total Bilirubin, mg/dL | 0.6 (0.4, 0.7) | 0.7 (0.4, 0.75) | 0.5 (0.3, 0.63) | 0.15 |
| Creatine Kinase, U/L | 94 (45.5, 170.3) | 135.5 (70.3, 178.8) | 54.5 (18.8, 194.5) | 0.24 |
| AST, U/L | 30 (23, 53) | 27 (21.5, 44) | 33.5 (25.8, 82.3) | 0.43 |
| ALT, U/L | 46 (23, 92) | 41 (19, 144.4) | 74 (28.3, 105) | 0.24 |
| **LD, U/L** | **270 (217, 415)** | **239 (208.5, 300)** | **490 (268.8, 650.8)** | **0.01** |
| ALP (IFCC), U/L | 66.0 ± 15.95 | 65.9 ± 16.26 | 66 ±16.79 | 0.99 |
| Creatinine, mg/dL | 0.8 (0.7, 0.97) | 0.8 (0.7, 0.96) | 0.83 (0.67, 0.99) | 0.69 |

| | | | | |
|---|---|---|---|---|
| **BUN, mg/dL** | **17 (12, 22)** | **14 (10.5, 27.4)** | **29 (16.8, 40.5)** | **0.05** |
| Uric Acid, mg/dL | 4.41 ± 1.85 | 4.68 ± 2.03 | 3.7 ± 1.17 | 0.33 |
| Na, mmol/L | 138 (136, 140) | 138 (137, 140) | 136 (132.3, 143.3) | 0.12 |
| Cl, mmol/L | 101 (99, 104) | 101 (98.5, 103) | 103 (100.3, 106.8) | 0.17 |
| K, mmol/L | 4.12 ± 0.47 | 4.0 ± 0.43 | 4.37 ± 0.48 | 0.12 |
| **Ca, mg/L** | **8.45 ± 0.55** | **8.73 ± 0.56** | **8.13 ± 0.14** | **0.04** |
| CRP, mg/dL | 0.64 (0.19, 3.05) | 0.64 (0.23, 2.93) | 1.27 (0.11, 5.15) | 0.86 |
| HbA1c, % | 5.9 (5.6, 6.1) | 5.8 (5.6, 5.98) | 6.3 (5.75, 8.3) | 0.07 |
| PT-INR | 0.97 (0.93, 1.02) | 0.99 (0.95, 1.03) | 0.95 (0.91, 1.00) | 0.20 |
| APTT, sec | 34.5 (27.9, 37.5) | 33.3 (27.5, 37.9) | 36.1 (29.6, 37.8) | 0.69 |
| Fibrinogen, mg/dL | 397.9 ± 115.0 | 385.5 ± 92.1 | 424.8 ± 161.3 | 0.50 |
| **D-dimer, ug/mL** | **0.6 (0, 0.9)** | **0.4 (0, 0.75)** | **0.9 (0.7, 8.58)** | **0.02** |
| Data are expressed as the mean ± SD for normally distributed data or median (IQR) for nonnormally distributed data. Statistical analysis was performed by using unpaired Student t-test and Wilcoxon rank-sum test. Noncritical and Critical groups were included in this comparison. Statistical significance was accepted as *P* < 0.05. Data in bold are statistically significant. | | | | |
| Abbreviations: AST, aspartate aminotransferase; ALT, alanine aminotransferase; LD, lactate dehydrogenase; ALP, alkaline phosphatase; BUN, blood urea nitrogen; Na, serum sodium; Cl, serum chloride; K, serum potassium; Ca, serum calcium; CRP: C-reactive protein; HbA1c, hemoglobin A1c; PT-INR, prothrombin time- international normalized ratio; APTT, activated partial thromboplastin time | | | | |

| **Supplementary Table 4. Comparison of serum biomarkers between healthy control and COVID-19 patients** | | | |
|---|---|---|---|
| | **Healthy control (*n* = 6)** | **COVID-19 (*n* = 19)** | ***P* value** |
| **IL-18, pg/ml** | **174.6 ± 74.30** | **402.0 ± 133.5** | **<0.001** |
| **TNF-α, pg/ml** | **1.68 (0.072, 6.24)** | **9.37 (7.93, 11.53)** | **<0.001** |
| **VEGF, pg/ml** | **70.1 (13.5, 787.0)** | **402.9 (266.7, 787.0)** | **<0.001** |
| ADAMTS13, ng/ml | 800.2 ± 114.13 | 749.7 ± 135.9 | 0.49 |
| ICAM-1, ng/ml | 172.2 ± 29.02 | 224.3 ± 84.3 | 0.18 |

Data are expressed as the mean ± SD for normally distributed data or median (IQR) for nonnormally distributed data. Statistical analysis was performed by using unpaired Student t-test and Wilcoxon rank-sum test. Statistical significance was accepted as $P < 0.05$. Data in bold are statistically significant.

**Supplementary Table 5. Metabolites identified as targets by metabolomic analysis**

| ID | HMT DB† | | Relative area | | | | | | Comparative Analysis | | |
|---|---|---|---|---|---|---|---|---|---|---|---|
| | Compound name | Pathway Label | Noncritical (n=5) | | Critical 1 acute phase (n=3) | | Critical 2 recovery phase (n=2) | | Noncritical (n=5) vs Critical 1 (acute phase, n=3) | | |
| | | | Mean | S.D. | Mean | S.D. | Mean | S.D. | Ratio ¶ | p-value‖ | |
| A_0082 | Indole-3-propionic acid | Indole-3-propionic acid | 1.0E-04 | 7.8E-06 | 4.1E-05 | 1.6E-05 | 2.6E-05 | N.A. | 2.5 | 0.013 | * |
| C_0068 | Asn | Asn | 3.1E-04 | 2.2E-04 | 1.9E-03 | 2.9E-04 | 7.2E-04 | N.A. | 0.2 | 0.029 | * |
| C_0081 | 1-Methyl-4-imidazoleacetic acid | MIA | 1.3E-03 | 8.3E-04 | 1.6E-04 | 6.3E-05 | 5.9E-04 | N.A. | 7.9 | 0.039 | * |
| A_0011 | Glyceric acid | Glyceric acid | 7.6E-04 | 2.8E-04 | 3.5E-04 | 1.3E-04 | 2.1E-04 | N.A. | 2.2 | 0.055 | |

| | | | | | | | | | | | |
|---|---|---|---|---|---|---|---|---|---|---|---|
| A_0073 | Homovanillic acid Hydroxyphenyllactic acid | HVA Hydroxyphenyllactic acid | 7.4E-05 | 1.7E-05 | 2.6E-04 | 1.1E-04 | 1.7E-03 | N.A. | 0.3 | 0.097 | |
| C_0056 | *N*-Methylproline | N-Methylproline | 3.0E-04 | 9.3E-05 | 1.6E-04 | 6.6E-05 | 2.2E-04 | N.A. | 1.9 | 0.106 | |
| C_0135 | Gly-Asp | Gly-Asp | 4.5E-05 | 1.7E-05 | 9.3E-05 | 7.9E-06 | N.A. | N.A. | 0.5 | 0.117 | |
| A_0118 | *N*-Acetylneuraminic acid | NeuNAc | 9.6E-04 | 7.0E-04 | 2.3E-04 | 2.7E-04 | 9.1E-05 | 8.2E-05 | 4.1 | 0.126 | |
| A_0019 | 2-Hydroxyvaleric acid | 2-Hydroxyvaleric acid | 2.2E-04 | 1.2E-04 | 5.8E-04 | 2.7E-04 | 2.9E-04 | 3.2E-04 | 0.4 | 0.136 | |

| | | | | | | | | | | | |
|---|---|---|---|---|---|---|---|---|---|---|---|
| C_0041 | Homoserine | Homoserine | 2.8E-04 | 1.9E-04 | 9.2E-05 | 4.6E-05 | 6.2E-05 | 1.2E-05 | 3.0 | 0.143 | |
| C_0083 | XC0029<br>Stachydrine | XC0029<br>Stachydrine | 3.5E-04 | 2.9E-04 | 1.2E-04 | 7.1E-05 | 8.3E-05 | N.A. | 2.9 | 0.159 | |
| A_0006 | Lactic acid | Lactic acid | 9.0E-04 | 5.9E-04 | 3.5E-04 | 2.3E-05 | 4.3E-04 | 3.0E-04 | 2.6 | 0.161 | |
| C_0002 | Ethanolamine | Ethanolamine | 1.7E-03 | 1.4E-03 | 4.4E-04 | 4.0E-04 | 1.8E-04 | 1.7E-04 | 3.9 | 0.176 | |
| A_0043 | 3-Phenylpropionic acid | 3-Phenylpropionic acid | 5.0E-04 | 5.2E-04 | 1.3E-04 | 8.3E-05 | 2.6E-04 | 2.0E-04 | 4.0 | 0.180 | |
| C_0109 | 7-Methylguanine | 7-Methylguanine | 3.5E-04 | 1.5E-04 | 2.1E-04 | 8.5E-05 | 2.6E-04 | 2.7E-05 | 1.6 | 0.200 | |
| C_0073 | Adenine | Adenine | 1.6E-04 | 1.0E-04 | 5.9E-05 | 4.0E-05 | 5.2E-05 | N.A. | 2.8 | 0.206 | |
| C_0151 | *N*-Acetylgalactosamine<br>*N*-Acetylglucosamine<br>*N*-Acetylmannosamine | N-Acetylgalactosamine<br>GlcNAc<br>ManNAc | 3.7E-03 | 3.4E-03 | 1.0E-03 | 6.8E-04 | 4.9E-04 | 3.8E-04 | 3.7 | 0.212 | |
| C_0059 | *N*-Acetylputrescine | N-Acetylputrescine | 6.6E-03 | 5.7E-03 | 8.1E-04 | 6.9E-04 | 4.4E-03 | N.A. | 8.2 | 0.216 | |

| | | | | | | | | | | | |
|---|---|---|---|---|---|---|---|---|---|---|---|
| A_0050 | *m*-Ethoxybenzoic acid 3-Phenyllactic acid *p*-Methoxyphenylacetic acid | m-Ethoxybenzoic acid 3-Phenyllactic acid p-Methoxyphenylacetic acid | 7.4E-05 | 6.5E-05 | 2.7E-04 | 1.9E-04 | 3.2E-04 | N.A. | 0.3 | 0.222 | |
| C_0133 | $N^6$,$N^6$,$N^6$-Trimethyllysine | Trimethyllysine | 1.3E-04 | 6.4E-05 | 4.3E-04 | 3.2E-04 | 2.7E-04 | 3.3E-04 | 0.3 | 0.239 | |
| C_0134 | Homocitrulline | Homocitrulline | 1.2E-04 | 1.1E-05 | 9.2E-05 | 1.9E-05 | 1.4E-04 | N.A. | 1.3 | 0.244 | |
| C_0079 | Tyramine | Tyramine | 1.6E-04 | 9.0E-05 | 3.5E-03 | 3.7E-03 | 1.5E-03 | 1.9E-03 | 0.05 | 0.257 | |
| C_0054 | 4-Oxopyrrolidine-2-carboxylic acid | 4-Oxopyrrolidine-2-carboxylic acid | 5.0E-04 | 4.5E-04 | 1.9E-04 | 1.2E-04 | 1.9E-04 | 2.8E-05 | 2.6 | 0.264 | |
| C_0103 | $N^6$-Methyllysine | N6-Methyllysine | 6.7E-05 | 2.7E-05 | 1.8E-04 | 7.7E-05 | N.A. | N.A. | 0.4 | 0.264 | |
| C_0162 | Cytidine | Cytidine | 3.2E-04 | 2.6E-04 | 9.1E-05 | 7.9E-05 | 3.1E-05 | 1.8E-05 | 3.5 | 0.266 | |
| C_0074 | Hypoxanthine | Hypoxanthine | 5.5E-03 | 7.3E-03 | 1.3E-03 | 1.1E-03 | 7.7E-04 | 9.4E-05 | 4.3 | 0.267 | |

| | | | | | | | | | | | | |
|---|---|---|---|---|---|---|---|---|---|---|---|---|
| A_0016 | Succinic acid | Succinic acid | 1.6E-03 | 1.2E-03 | 6.1E-04 | 3.0E-04 | 8.0E-04 | 3.3E-04 | 2.7 | 0.272 | |
| C_0107 | 5-Hydroxylysine | 5-Hydroxylysine | 6.7E-05 | 2.4E-05 | 3.5E-04 | 1.9E-04 | 4.1E-05 | N.A. | 0.2 | 0.280 | |
| A_0008 | Isovaleric acid<br>DL-2-Methylbutyric Acid<br>Valeric acid | Isovaleric acid<br>2-Methylbutyric acid<br>Valeric acid | 1.3E-02 | 1.1E-02 | 2.4E-02 | 1.3E-02 | 3.2E-02 | 1.4E-02 | 0.5 | 0.285 | |
| A_0051 | 3-(4-Hydroxyphenyl)propionic acid<br>2-(4-Hydroxyphenyl)propionic acid<br>3-(3-Hydroxyphenyl)propionic acid | 3-(4-Hydroxyphenyl)propionic acid<br>2-(4-Hydroxyphenyl)propionic acid<br>3-(3-Hydroxyphenyl)propionic acid | 1.4E-03 | 9.3E-04 | 5.2E-04 | 5.1E-04 | 4.2E-04 | N.A. | 2.6 | 0.286 | |
| C_0169 | XC0089 | XC0089 | 9.8E-05 | 2.7E-05 | 6.4E-05 | 1.9E-05 | 7.9E-05 | 4.9E-05 | 1.5 | 0.289 | |
| C_0095 | Xanthine | Xanthine | 5.4E-03 | 8.3E-03 | 8.6E-04 | 5.8E-04 | 9.3E-04 | 9.1E-04 | 6.3 | 0.289 | |

| | | | | | | | | | | | |
|---|---|---|---|---|---|---|---|---|---|---|---|
| A_0027 | 2-Hydroxy-4-methylvaleric acid | 2-Hydroxy-4-methylvaleric acid | 3.1E-04 | 2.3E-04 | 6.6E-04 | 4.4E-04 | 3.6E-04 | 4.4E-04 | 0.5 | 0.311 | |
| C_0021 | Cadaverine | Cadaverine | 5.8E-03 | 7.7E-03 | 1.6E-02 | 1.3E-02 | 6.5E-04 | 9.0E-04 | 0.4 | 0.316 | |
| C_0012 | β-Ala | b-Ala | 7.4E-04 | 5.4E-04 | 2.7E-03 | 2.6E-03 | 9.2E-04 | 1.1E-03 | 0.3 | 0.318 | |
| C_0009 | Putrescine | Putrescine | 6.7E-03 | 5.6E-03 | 2.4E-03 | 2.6E-03 | 5.2E-03 | N.A. | 2.8 | 0.318 | |
| C_0040 | 2-Methylserine | 2-Methylserine | 1.1E-04 | 7.2E-05 | 1.7E-04 | 3.9E-05 | 5.0E-04 | N.A. | 0.7 | 0.318 | |
| C_0129 | $N^8$-Acetylspermidine | N8-Acetylspermidine | 1.6E-04 | 1.0E-04 | 7.3E-05 | 9.0E-05 | 2.6E-05 | N.A. | 2.2 | 0.325 | |
| C_0090 | Glu | Glu | 5.4E-02 | 6.0E-02 | 2.3E-02 | 1.6E-02 | 2.4E-02 | 2.7E-03 | 2.3 | 0.327 | |
| C_0033 | Uracil | Uracil | 2.6E-03 | 3.8E-03 | 3.6E-04 | 2.3E-04 | 4.6E-04 | N.A. | 7.2 | 0.327 | |
| C_0185 | His-Glu | His-Glu | 1.5E-05 | 8.4E-06 | 4.2E-05 | 2.4E-05 | N.A. | N.A. | 0.3 | 0.337 | |

| | | | | | | | | | | | | |
|---|---|---|---|---|---|---|---|---|---|---|---|---|
| C_0006 | Isopropanolamine | Isopropanolamine | 2.1E-04 | 1.8E-04 | 5.0E-04 | 4.0E-04 | 1.4E-04 | 3.9E-05 | 0.4 | 0.337 | |
| C_0128 | *N*[1]-Acetylspermidine | N1-Acetylspermidine | 9.7E-04 | 1.1E-03 | 3.0E-04 | 3.4E-04 | 4.0E-05 | N.A. | 3.2 | 0.337 | |
| C_0118 | Arg | Arg | 2.5E-03 | 3.0E-03 | 4.7E-03 | 2.8E-03 | 2.9E-03 | 3.0E-03 | 0.5 | 0.344 | |
| C_0085 | γ-Butyrobetaine | Actinine | 3.0E-03 | 2.4E-03 | 2.2E-02 | 2.7E-02 | 2.2E-02 | 4.7E-03 | 0.13 | 0.344 | |
| A_0136 | Cholic acid | Cholic acid | 2.6E-03 | 1.6E-03 | 1.1E-02 | 1.2E-02 | 4.3E-02 | 5.8E-02 | 0.2 | 0.349 | |
| C_0001 | Trimethylamine | Trimethylamine | 2.7E-03 | 1.8E-03 | 1.7E-03 | 5.7E-04 | 1.9E-03 | 3.0E-04 | 1.6 | 0.354 | |
| C_0136 | 2,6-Diaminopimelic acid | 2,6-Diaminopimelic acid | 5.7E-04 | 7.5E-04 | 6.1E-05 | 2.0E-05 | 4.2E-05 | N.A. | 9.3 | 0.363 | |
| C_0154 | *N*[1],*N*[8]-Diacetylspermidine | N1,N8-Diacetylspermidine | 7.3E-04 | 8.7E-04 | 1.4E-04 | 1.1E-04 | 7.3E-05 | 8.8E-07 | 5.1 | 0.364 | |
| A_0054 | Uric acid | Uric acid | 2.7E-04 | 3.7E-04 | 9.8E-05 | 2.3E-05 | 1.0E-04 | 6.1E-05 | 2.7 | 0.367 | |

| | | | | | | | | | | | |
|---|---|---|---|---|---|---|---|---|---|---|---|
| C_0180 | γ-Glu-Glu | g-Glu-Glu | 2.5E-04 | 3.3E-04 | 7.8E-05 | 4.2E-08 | 8.2E-05 | 4.7E-05 | 3.2 | 0.369 | |
| C_0025 | 3-Aminoisobutyric acid | 3-Aminoisobutyric acid | 1.1E-04 | 4.9E-05 | 2.2E-03 | 3.2E-03 | 1.3E-03 | 1.1E-03 | 0.05 | 0.374 | |
| C_0017 | Phenol | Phenol | 9.0E-04 | 1.6E-03 | 2.1E-04 | 5.5E-05 | 2.3E-04 | 2.4E-05 | 4.3 | 0.376 | |
| A_0095 | $N^2$-Acetylaminoadipic acid | N2-Acetylaminoadipic acid | 6.3E-05 | 4.5E-05 | 3.4E-05 | 1.4E-05 | 5.4E-05 | N.A. | 1.9 | 0.378 | |
| A_0022 | 5-Oxoproline | Oxoproline | 2.9E-04 | 4.4E-04 | 6.6E-05 | 1.3E-05 | 1.9E-04 | 1.1E-05 | 4.5 | 0.379 | |
| C_0094 | Guanine | Guanine | 1.9E-04 | 1.7E-04 | 9.3E-05 | 8.5E-05 | 4.2E-05 | 1.4E-05 | 2.0 | 0.385 | |
| C_0016 | Glycerol | Glycerol | 3.8E-02 | 4.9E-02 | 6.8E-02 | 4.1E-02 | 8.7E-02 | 7.0E-02 | 0.6 | 0.390 | |
| C_0097 | 4-(β-Acetylaminoethyl)imidazole | 4-(b-Acetylaminoethyl)imidazole | 5.5E-03 | 1.1E-02 | 1.6E-04 | 1.1E-04 | 5.6E-05 | 2.0E-06 | 34 | 0.392 | |
| C_0132 | Gly-Leu | Gly-Leu | 4.5E-04 | 2.1E-04 | 7.7E-04 | 5.1E-04 | 2.9E-04 | 1.7E-04 | 0.6 | 0.392 | |

| | | | | | | | | | | | | |
|---|---|---|---|---|---|---|---|---|---|---|---|---|
| A_0009 | 2-Hydroxybutyric acid | 2-HBA | 1.5E-04 | 6.0E-05 | 2.3E-04 | 8.0E-05 | 2.7E-04 | N.A. | 0.7 | 0.394 | |
| C_0108 | Methionine sulfoxide | Methionine sulfoxide | 4.2E-03 | 7.9E-03 | 3.1E-04 | 1.9E-04 | 2.2E-04 | 1.9E-05 | 14 | 0.399 | |
| A_0002 | Propionic acid | Propionic acid | 2.4E-02 | 1.9E-02 | 3.4E-02 | 1.4E-02 | 2.6E-02 | 1.3E-04 | 0.7 | 0.409 | |
| C_0184 | Guanosine | Guanosine | 1.5E-04 | 9.2E-05 | 2.8E-04 | 2.3E-04 | 9.4E-05 | 3.6E-05 | 0.5 | 0.410 | |
| A_0102 | Pantothenic acid | Pantothenic acid | 2.1E-04 | 2.6E-04 | 1.0E-03 | 1.4E-03 | 9.4E-04 | 1.1E-03 | 0.2 | 0.422 | |
| A_0092 | Syringic acid | Syringic acid | 1.9E-04 | 2.6E-04 | 4.2E-05 | 5.3E-06 | 4.0E-05 | N.A. | 4.6 | 0.424 | |
| A_0014 | Hexanoic acid | Hexanoic acid | 3.2E-04 | 1.9E-04 | 4.2E-03 | 6.8E-03 | 8.3E-03 | 1.1E-02 | 0.08 | 0.426 | |
| C_0067 | Gly-Gly | Gly-Gly | 8.9E-05 | 4.7E-05 | 1.3E-04 | 4.2E-05 | N.A. | N.A. | 0.7 | 0.432 | |
| C_0070 | Asp | Asp | 1.7E-02 | 2.5E-02 | 6.8E-03 | 4.8E-03 | 5.6E-03 | 7.9E-07 | 2.4 | 0.435 | |

| | | | | | | | | | | | | |
|---|---|---|---|---|---|---|---|---|---|---|---|---|
| C_0159 | Cystine | Cystine | 5.2E-05 | 5.1E-05 | 8.0E-05 | 4.5E-06 | 2.8E-05 | N.A. | 0.6 | 0.440 | |
| C_0029 | Diethanolamine | Diethanolamine | 1.2E-04 | 1.4E-04 | 6.4E-05 | 8.7E-06 | 4.4E-05 | N.A. | 1.9 | 0.446 | |
| C_0150 | *N*-Acetylglucosylamine | N-Acetylglucosylamine | 7.8E-05 | 5.0E-05 | 3.7E-05 | 1.8E-06 | 9.1E-05 | N.A. | 2.1 | 0.447 | |
| A_0045 | 3-Hydroxyphenylacetic acid<br>*p*-Hydroxyphenylacetic acid<br>*p*-Anisic acid | 3-Hydroxyphenylacetic acid<br>4-HPAA<br>p-Anisic acid | 7.0E-04 | 5.5E-04 | 4.5E-04 | 2.3E-04 | 8.2E-04 | N.A. | 1.6 | 0.453 | |
| C_0172 | γ-Glu-Ile<br>γ-Glu-Leu | g-Glu-Ile<br>g-Glu-Leu | 1.4E-04 | 1.3E-04 | 6.7E-05 | 1.9E-05 | N.A. | N.A. | 2.1 | 0.457 | |
| C_0037 | 5-Aminovaleric acid | 5-Aminovaleric acid | 5.1E-03 | 6.1E-03 | 1.8E-02 | 2.5E-02 | 3.4E-03 | 2.7E-03 | 0.3 | 0.459 | |
| C_0003 | XC0001 | XC0001 | 6.6E-04 | 9.5E-04 | 3.1E-04 | 1.3E-04 | 6.4E-04 | 2.2E-04 | 2.1 | 0.460 | |
| C_0007 | Piperidine | Piperidine | 2.0E-04 | 1.0E-04 | 7.8E-04 | 1.1E-03 | 1.0E-04 | 7.0E-05 | 0.3 | 0.461 | |

| | | | | | | | | | | | |
|---|---|---|---|---|---|---|---|---|---|---|---|
| A_0049 | Terephthalic acid | Terephthalic acid | 1.4E-04 | 1.3E-04 | 8.8E-05 | 3.3E-05 | 1.0E-04 | 9.1E-06 | 1.5 | 0.467 | |
| C_0089 | Isoglutamic acid | Isoglutamic acid | 1.0E-03 | 1.1E-03 | 6.7E-03 | 1.1E-02 | 1.0E-02 | 1.1E-02 | 0.2 | 0.468 | |
| A_0029 | *p*-Toluic acid<br>*o*-Toluic acid<br>*m*-Toluic acid | p-Toluic acid<br>o-Toluic acid<br>m-Toluic acid | 1.3E-03 | 5.3E-04 | 2.2E-03 | 1.9E-03 | 3.7E-03 | 3.0E-04 | 0.6 | 0.470 | |
| C_0005 | Gly | Gly | 1.8E-02 | 3.3E-02 | 5.7E-03 | 3.4E-03 | 3.7E-03 | 1.3E-03 | 3.1 | 0.471 | |
| C_0123 | Glucosamine | Glucosamine | 1.7E-04 | 1.7E-04 | 8.2E-05 | 4.9E-05 | 4.5E-05 | 6.6E-06 | 2.0 | 0.475 | |
| C_0145 | Carboxymethyllysine | Carboxymethyllysine | 7.1E-05 | 7.0E-05 | 2.2E-03 | 2.8E-03 | 8.3E-04 | 9.9E-04 | 0.03 | 0.475 | |
| C_0182 | Saccharopine | Saccharopine | 4.3E-05 | 1.9E-06 | 1.1E-04 | 1.3E-04 | 4.5E-05 | 2.5E-05 | 0.4 | 0.489 | |
| C_0084 | 4-Guanidinobutyric acid | 4-GBA | 1.2E-04 | 1.0E-04 | 7.0E-05 | 2.9E-05 | N.A. | N.A. | 1.7 | 0.493 | |
| A_0025 | *N*-Acetylalanine | N-Acetylalanine | 6.2E-05 | 3.3E-05 | 4.7E-05 | 7.2E-06 | 3.0E-05 | N.A. | 1.3 | 0.511 | |

| | | | | | | | | | | | |
|---|---|---|---|---|---|---|---|---|---|---|---|
| C_0111 | Pyridoxamine | Pyridoxamine | 8.5E-05 | 3.1E-05 | 1.2E-04 | 8.4E-05 | 1.6E-04 | 2.2E-05 | 0.7 | 0.512 | |
| C_0061 | Hydroxyproline | Hydroxyproline | 1.8E-04 | 1.4E-04 | 2.6E-03 | 3.6E-03 | N.A. | N.A. | 0.07 | 0.518 | |
| A_0039 | 2-Hydroxyglutaric acid | 2-Hydroxyglutaric acid | 2.8E-04 | 7.2E-05 | 3.2E-04 | 3.4E-05 | 6.5E-03 | N.A. | 0.9 | 0.518 | |
| C_0063 | Creatine | Creatine | 4.4E-04 | 7.1E-04 | 4.5E-03 | 6.1E-03 | 2.5E-04 | N.A. | 0.10 | 0.521 | |
| C_0101 | XC0145<br>Ala-Ala | XC0145<br>Ala-Ala | 1.9E-03 | 2.4E-03 | 9.9E-04 | 4.1E-04 | 5.5E-04 | 2.9E-04 | 1.9 | 0.522 | |
| C_0052 | Imidazole-4-acetic acid | Imidazole-4-acetic acid | 1.7E-04 | 9.6E-05 | 7.0E-04 | 8.3E-04 | 1.0E-04 | N.A. | 0.2 | 0.531 | |
| C_0113 | 1-Methylhistidine<br>3-Methylhistidine | 1-Methylhistidine<br>3-Methylhistidine | 4.6E-04 | 3.3E-04 | 2.7E-04 | 3.8E-04 | 9.2E-05 | N.A. | 1.7 | 0.537 | |
| A_0085 | *N*-Acetylmethionine | N-Acetylmethionine | 3.9E-05 | 3.0E-05 | 2.5E-05 | 1.2E-05 | 2.5E-05 | 1.4E-05 | 1.5 | 0.540 | |
| C_0119 | Citrulline | Citrulline | 9.6E-03 | 1.6E-02 | 4.8E-03 | 2.7E-03 | 2.8E-03 | 2.9E-04 | 2.0 | 0.546 | |

| | | | | | | | | | | | |
|---|---|---|---|---|---|---|---|---|---|---|---|
| C_0080 | 1*H*-Imidazole-4-propionic acid | 1H-Imidazole-4-propionic acid | 1.5E-03 | 4.5E-04 | 4.0E-03 | 4.1E-03 | 9.3E-03 | N.A. | 0.4 | 0.549 | |
| C_0153 | 2'-Deoxycytidine | dCyt | 2.6E-04 | 3.7E-04 | 1.3E-04 | 1.7E-04 | 4.6E-05 | 3.0E-05 | 2.1 | 0.560 | |
| C_0011 | Ala | Ala | 4.2E-02 | 6.8E-02 | 2.3E-02 | 1.1E-02 | 1.4E-02 | 1.3E-03 | 1.9 | 0.564 | |
| C_0088 | Lys | Lys | 2.6E-02 | 4.1E-02 | 1.4E-02 | 7.1E-03 | 9.2E-03 | 2.1E-04 | 1.8 | 0.575 | |
| C_0066 | Alloisoleucine | Alloisoleucine | 5.4E-03 | 1.0E-02 | 2.1E-03 | 2.6E-03 | 5.4E-04 | 5.7E-04 | 2.5 | 0.580 | |
| C_0130 | $N^6$-Acetyllysine | N6-Acetyllysine | 1.1E-04 | 8.2E-05 | 1.6E-04 | 1.0E-04 | 9.6E-05 | 7.2E-06 | 0.7 | 0.585 | |
| C_0055 | Pipecolic acid | Pipecolic acid | 2.2E-03 | 1.9E-03 | 4.6E-03 | 6.5E-03 | 5.0E-03 | 7.7E-04 | 0.5 | 0.593 | |
| C_0161 | Thymidine | Thymidine | 4.1E-04 | 2.1E-04 | 3.4E-04 | 1.5E-04 | 4.9E-04 | 1.2E-04 | 1.2 | 0.606 | |
| C_0181 | Glu-Glu | Glu-Glu | 9.7E-05 | 8.4E-05 | 1.6E-04 | 1.8E-04 | 5.8E-05 | N.A. | 0.6 | 0.607 | |

| | | | | | | | | | | | | |
|---|---|---|---|---|---|---|---|---|---|---|---|---|
| C_0140 | SDMA | SDMA | 1.5E-04 | 7.8E-05 | 2.2E-04 | 1.6E-04 | 1.5E-04 | N.A. | 0.7 | 0.608 | |
| C_0173 | γ-Glu-Asn | g-Glu-Asn | 2.3E-05 | 7.1E-06 | 2.7E-05 | 5.4E-06 | 4.5E-05 | N.A. | 0.9 | 0.608 | |
| C_0069 | Ornithine | Ornithine | 3.5E-03 | 5.2E-03 | 2.2E-03 | 1.8E-03 | 7.3E-04 | 2.1E-04 | 1.6 | 0.615 | |
| A_0080 | Kynurenic acid | Kynurenic acid | 2.8E-05 | 1.2E-05 | 2.2E-05 | 1.1E-05 | 4.2E-05 | 1.9E-05 | 1.3 | 0.620 | |
| **C_0144** | **Trp** | **Trp** | **2.8E-03** | **4.2E-03** | **1.7E-03** | **3.2E-04** | **7.7E-04** | **2.6E-04** | **1.7** | **0.621** | |
| A_0084 | 5-Hydroxyindoleacetic acid | 5-Hydroxy-IAA | 5.8E-05 | 3.3E-05 | 4.2E-05 | 3.4E-05 | N.A. | N.A. | 1.4 | 0.624 | |
| C_0035 | Pro | Pro | 1.6E-02 | 2.8E-02 | 3.1E-02 | 4.4E-02 | 3.7E-03 | 2.0E-03 | 0.5 | 0.633 | |
| C_0126 | Tyr | Tyr | 1.1E-02 | 1.9E-02 | 7.2E-03 | 1.7E-03 | 4.9E-03 | 2.9E-03 | 1.6 | 0.641 | |
| C_0039 | Thr | Thr | 7.7E-03 | 1.0E-02 | 5.3E-03 | 2.7E-03 | 3.6E-03 | 1.1E-03 | 1.5 | 0.644 | |

| | | | | | | | | | | | |
|---|---|---|---|---|---|---|---|---|---|---|---|
| C_0092 | Met | Met | 6.3E-03 | 1.1E-02 | 3.9E-03 | 1.9E-03 | 2.2E-03 | 2.5E-04 | 1.6 | 0.648 | |
| C_0163 | Uridine | Uridine | 3.6E-04 | 2.2E-04 | 3.1E-04 | 8.2E-06 | 1.8E-04 | 1.4E-05 | 1.2 | 0.652 | |
| C_0064 | Ile | Ile | 3.7E-02 | 6.3E-02 | 2.3E-02 | 1.1E-02 | 7.4E-03 | 9.8E-05 | 1.6 | 0.653 | |
| C_0106 | Carnitine | Carnitine | 5.6E-04 | 5.7E-04 | 7.8E-04 | 6.3E-04 | 1.2E-03 | 1.0E-03 | 0.7 | 0.657 | |
| A_0062 | Isovalerylalanine-1<br>N-Acetylleucine-1 | Isovalerylalanine<br>N-Acetylleucine | 1.1E-04 | 4.5E-05 | 1.6E-04 | 1.9E-04 | 7.9E-05 | 3.6E-05 | 0.7 | 0.660 | |
| C_0028 | Ser | Ser | 7.9E-03 | 1.2E-02 | 5.2E-03 | 2.5E-03 | 3.2E-03 | 4.9E-04 | 1.5 | 0.661 | |
| C_0176 | 2'-Deoxyguanosine | dGuanosine | 2.2E-04 | 1.2E-04 | 3.6E-04 | 4.6E-04 | 1.0E-04 | 4.3E-05 | 0.6 | 0.664 | |
| C_0026 | GABA | GABA | 4.7E-04 | 3.9E-04 | 7.4E-04 | 9.3E-04 | 2.7E-04 | 5.4E-05 | 0.6 | 0.673 | |
| C_0023 | 2-Aminoisobutyric acid<br>2-Aminobutyric acid | 2-Aminoisobutyric acid<br>2-Aminobutyric acid | 8.9E-03 | 8.4E-03 | 6.5E-03 | 6.0E-03 | 1.8E-03 | 2.4E-04 | 1.4 | 0.673 | |

| | | | | | | | | | | | |
|---|---|---|---|---|---|---|---|---|---|---|---|
| A_0030 | *m*-Hydroxybenzoic acid<br>*p*-Hydroxybenzoic acid | m-Hydroxybenzoic acid<br>p-Hydroxybenzoic acid | 7.0E-05 | 1.1E-05 | 7.6E-05 | 1.6E-05 | 7.3E-05 | N.A. | 0.9 | 0.677 | |
| C_0098 | His | His | 1.9E-03 | 2.2E-03 | 2.5E-03 | 1.6E-03 | 1.8E-03 | 6.6E-04 | 0.8 | 0.679 | |
| A_0018 | 5-Hydroxypentanoic acid | 5-Hydroxypentanoic acid | 1.2E-03 | 1.5E-03 | 6.1E-04 | 6.1E-04 | 5.0E-05 | N.A. | 2.0 | 0.681 | |
| A_0078 | XA0017 | XA0017 | 1.3E-04 | 8.8E-05 | 8.9E-05 | 9.2E-05 | 2.3E-05 | N.A. | 1.4 | 0.686 | |
| A_0141 | Glycocholic acid | Glycocholic acid | 4.3E-05 | 3.4E-06 | 3.8E-05 | 1.4E-05 | 5.6E-05 | 1.2E-05 | 1.1 | 0.686 | |
| C_0048 | Nicotinic acid | Nicotinic acid | 1.8E-03 | 1.1E-03 | 1.5E-03 | 1.3E-03 | 9.6E-04 | 2.3E-04 | 1.2 | 0.713 | |
| C_0157 | Ser-Glu | Ser-Glu | 1.4E-04 | 7.8E-05 | 1.7E-04 | 1.4E-04 | 6.8E-05 | 1.9E-05 | 0.8 | 0.720 | |
| C_0027 | Choline | Choline | 2.0E-03 | 2.0E-03 | 2.3E-03 | 7.6E-04 | 1.7E-03 | 2.0E-03 | 0.8 | 0.722 | |
| C_0086 | Spermidine | Spermidine | 5.9E-04 | 4.3E-04 | 6.9E-04 | 3.6E-04 | 4.8E-04 | N.A. | 0.8 | 0.726 | |

| | | | | | | | | | | | | |
|---|---|---|---|---|---|---|---|---|---|---|---|---|
| A_0026 | Glutaric acid<br>Methylsuccinic acid | Glutaric acid<br>Methylsuccinic acid | 1.2E-03 | 5.4E-04 | 9.5E-04 | 1.0E-03 | 1.0E-03 | 4.5E-04 | 1.3 | 0.738 | |
| A_0010 | 3-Hydroxybutyric acid | 3-HBA | 3.0E-04 | 3.2E-05 | 2.3E-04 | 2.3E-04 | N.A. | N.A. | 1.3 | 0.740 | |
| A_0028 | Malic acid | Malic acid | 2.1E-03 | 1.7E-03 | 2.4E-03 | 8.0E-04 | 2.1E-03 | 1.9E-04 | 0.9 | 0.743 | |
| C_0105 | *O*-Acetylhomoserine<br>2-Aminoadipic acid | O-Acetylhomoserine<br>2-Aminoadipic acid | 1.3E-04 | 7.2E-05 | 1.1E-04 | 4.2E-05 | 1.2E-04 | 3.9E-05 | 1.1 | 0.754 | |
| C_0139 | *N*-Acetylhistidine | N-Acetylhistidine | 1.3E-04 | 8.9E-05 | 1.0E-04 | 7.2E-05 | 2.6E-05 | N.A. | 1.2 | 0.757 | |
| C_0065 | Leu | Leu | 5.4E-02 | 8.4E-02 | 4.3E-02 | 2.5E-02 | 1.6E-02 | 4.3E-03 | 1.3 | 0.784 | |
| A_0075 | 4-Pyridoxic acid | 4-Pyridoxic acid | 8.0E-05 | 3.3E-05 | 9.1E-05 | 5.8E-05 | 1.4E-04 | 4.9E-05 | 0.9 | 0.791 | |
| C_0110 | Phe | Phe | 2.2E-02 | 3.7E-02 | 1.8E-02 | 7.2E-03 | 9.4E-03 | 4.2E-03 | 1.2 | 0.805 | |
| A_0116 | *N*-Acetylmuramic acid | N-Acetylmuramic acid | 1.8E-04 | 2.9E-04 | 1.4E-04 | 9.5E-05 | 3.1E-04 | 3.4E-04 | 1.3 | 0.813 | |

| | | | | | | | | | | | |
|---|---|---|---|---|---|---|---|---|---|---|---|
| C_0170 | XC0154 | XC0154 | 1.2E-03 | 7.3E-04 | 1.1E-03 | 2.6E-04 | 1.3E-03 | 5.5E-05 | 1.1 | 0.819 | |
| C_0191 | Arg-Glu | Arg-Glu | 7.3E-05 | 5.6E-05 | 6.4E-05 | 4.2E-05 | 3.4E-05 | 9.7E-06 | 1.1 | 0.834 | |
| C_0146 | Lipoamide | Lipoamide | 1.2E-04 | 1.0E-04 | 9.7E-05 | 1.1E-04 | 2.6E-05 | N.A. | 1.2 | 0.849 | |
| C_0178 | Inosine | Inosine | 6.5E-04 | 7.6E-04 | 7.3E-04 | 4.5E-04 | 2.9E-04 | 1.1E-04 | 0.9 | 0.849 | |
| C_0124 | Mannosamine | Mannosamine | 1.4E-04 | 9.9E-05 | 1.6E-04 | 1.2E-04 | 8.5E-05 | N.A. | 0.9 | 0.858 | |
| C_0183 | γ-Glu-Met | g-Glu-Met | 4.4E-05 | 3.5E-05 | 4.0E-05 | 1.6E-05 | 5.6E-05 | 3.1E-05 | 1.1 | 0.870 | |
| A_0005 | Butyric acid<br>Isobutyric acid | Butyric acid<br>Isobutyric acid | 3.1E-02 | 3.0E-02 | 3.4E-02 | 8.9E-03 | 3.1E-02 | 3.9E-03 | 0.9 | 0.871 | |
| C_0166 | Octopine | Octopine | 2.3E-04 | 7.7E-05 | 2.2E-04 | 1.4E-04 | 1.3E-04 | N.A. | 1.1 | 0.882 | |
| A_0035 | 4-Acetamidobutanoic acid | 4-Acetamidobutanoic acid | 6.7E-05 | 2.0E-05 | 6.4E-05 | 2.3E-05 | 7.7E-05 | 4.0E-05 | 1.1 | 0.886 | |

| | | | | | | | | | | | |
|---|---|---|---|---|---|---|---|---|---|---|---|
| A_0081 | *N*-Acetylglutamic acid | N-AcGlu | 3.8E-04 | 1.1E-04 | 3.5E-04 | 3.3E-04 | 2.2E-04 | 1.9E-05 | 1.1 | 0.900 | |
| C_0087 | Gln | Gln | 3.4E-03 | 3.0E-03 | 3.2E-03 | 1.4E-03 | 1.8E-03 | 5.2E-04 | 1.1 | 0.905 | |
| C_0038 | Val | Val | 4.0E-02 | 5.7E-02 | 3.7E-02 | 2.7E-02 | 1.2E-02 | 2.0E-03 | 1.1 | 0.912 | |
| A_0060 | Glycerol 3-phosphate | G3P | 1.5E-04 | 4.1E-05 | 1.7E-04 | 2.0E-04 | 3.0E-04 | N.A. | 0.9 | 0.918 | |
| C_0022 | *N*,*N*-Dimethylglycine | DMG | 1.0E-04 | 5.9E-05 | 9.8E-05 | 2.9E-05 | N.A. | N.A. | 1.0 | 0.945 | |
| A_0072 | 1-Methyluric acid | 1-Methyluric acid | 9.6E-05 | 6.5E-05 | 9.3E-05 | 5.1E-05 | 8.5E-05 | 1.8E-05 | 1.0 | 0.959 | |
| C_0014 | Oxalic acid | Oxalic acid | 8.3E-05 | 3.6E-05 | 8.1E-05 | 5.5E-05 | 4.4E-05 | 3.3E-06 | 1.0 | 0.972 | |
| C_0131 | *N*-Acetyllysine | N-Acetyllysine | 1.2E-03 | 7.1E-04 | 1.2E-03 | 9.4E-04 | 1.1E-03 | 4.7E-04 | 1.0 | 0.978 | |
| C_0175 | Thiamine | Thiamine | 3.3E-04 | 5.2E-04 | 3.2E-04 | 2.9E-04 | 2.7E-04 | 1.5E-04 | 1.0 | 0.982 | |

| | | | | | | | | | | | | |
|---|---|---|---|---|---|---|---|---|---|---|---|---|
| A_0012 | Fumaric acid | Fumaric acid | 3.6E-04 | 1.3E-04 | 3.6E-04 | 2.7E-04 | 2.1E-04 | 4.5E-05 | 1.0 | 0.984 | |
| C_0116 | *N*-Acetylornithine | N-AcOrn | 6.4E-04 | 5.2E-04 | 6.5E-04 | 4.7E-04 | 1.9E-04 | 1.6E-05 | 1.0 | 0.989 | |
| A_0023 | 4-Methyl-2-oxovaleric acid<br>3-Methyl-2-oxovaleric acid<br>2-Oxohexanoic acid | 2-Oxoleucine<br>2K3MVA<br>2-Oxohexanoic acid | 9.4E-04 | 1.3E-03 | 9.3E-04 | 1.0E-03 | 5.1E-04 | 3.6E-04 | 1.0 | 0.996 | |
| C_0199 | *S*-Adenosylmethionine | SAM | 4.3E-05 | 2.2E-05 | 4.3E-05 | 4.2E-05 | N.A. | N.A. | 1.0 | 0.999 | |
| C_0018 | 1-Aminocyclopropane-1-carboxylic acid | 1-Aminocyclopropane-1-carboxylic acid | 4.3E-05 | N.A. | N.A. | N.A. | N.A. | N.A. | 1< | N.A. | |
| C_0075 | 1-Methylnicotinamide | 1-Methylnicotinamide | N.A. | N.A. | 2.8E-05 | 1.3E-05 | 5.7E-05 | N.A. | <1 | N.A. | |
| C_0168 | 2'-Deoxyinosine | 2'-Deoxyinosine | 2.9E-04 | 1.7E-04 | 3.4E-04 | N.A. | 1.6E-04 | N.A. | 0.9 | N.A. | |
| C_0045 | 2-Amino-2-(hydroxymethyl)-1,3-propanediol | 2-Amino-2-(hydroxymethyl)-1,3-propanediol | 3.5E-03 | 6.0E-04 | 1.1E-03 | N.A. | 9.1E-05 | 5.9E-05 | 3.1 | N.A. | |

| | | | | | | | | | | | | |
|---|---|---|---|---|---|---|---|---|---|---|---|---|
| A_0020 | 2-Aminoethylphosphonic acid | 2-Aminoethylphosphonic acid | 7.5E-05 | 8.3E-05 | 3.8E-04 | N.A. | N.A. | N.A. | 0.2 | N.A. | |
| A_0099 | 2-Deoxyribose 1-phosphate | dR1P | 6.4E-05 | 2.4E-06 | N.A. | N.A. | 4.1E-04 | N.A. | 1< | N.A. | |
| A_0036 | 2-Oxoglutaric acid | 2-OG | 6.6E-04 | N.A. | 6.3E-04 | N.A. | 4.3E-04 | N.A. | 1.0 | N.A. | |
| A_0013 | 2-Oxoisovaleric acid | 2-KIV | 4.6E-04 | N.A. | 3.7E-04 | N.A. | 7.6E-05 | N.A. | 1.3 | N.A. | |
| C_0046 | 2-Phenylethylamine | Phenylethylamine | 9.4E-05 | N.A. | 1.6E-04 | 7.1E-05 | 2.8E-04 | 2.9E-04 | 0.6 | N.A. | |
| A_0150 | 3'-Dephospho CoA | Dephospho-CoA | 3.8E-05 | N.A. | N.A. | N.A. | N.A. | N.A. | 1< | N.A. | |
| A_0074 | 3,4-Dihydroxyhydrocinnamic acid | 3,4-Dihydroxyhydrocinnamic acid | 3.4E-04 | 3.8E-04 | 2.2E-04 | N.A. | N.A. | N.A. | 1.5 | N.A. | |
| A_0056 | 3,4-Dihydroxyphenylacetic acid | DOPAC | 3.4E-05 | N.A. | 6.8E-05 | N.A. | N.A. | N.A. | 0.5 | N.A. | |

| | | | | | | | | | | | |
|---|---|---|---|---|---|---|---|---|---|---|---|
| C_0024 | 3-Aminobutyric acid | 3-Aminobutyric acid | 3.3E-04 | N.A. | 6.4E-04 | 8.2E-04 | 2.6E-04 | 1.4E-04 | 0.5 | N.A. | |
| C_0015 | 3-Aminopropane-1,2-diol | 3-Aminopropane-1,2-diol | 3.7E-05 | N.A. | 4.4E-05 | 1.4E-05 | 5.7E-05 | N.A. | 0.8 | N.A. | |
| C_0062 | 3-Guanidinopropionic acid | 3-Guanidinopropionic acid | 2.5E-05 | N.A. | 3.5E-05 | 7.4E-06 | 3.4E-05 | N.A. | 0.7 | N.A. | |
| A_0007 | 3-Hydroxypropionic acid | b-Lactate | 2.6E-04 | 1.7E-04 | 1.5E-04 | N.A. | 3.3E-04 | 2.8E-04 | 1.7 | N.A. | |
| C_0093 | 3-Methyladenine | 3-Methyladenine | 5.1E-05 | N.A. | N.A. | N.A. | N.A. | N.A. | 1< | N.A. | |
| A_0077 | 3-Phosphoglyceric acid | 3-PG | 1.7E-04 | N.A. | N.A. | N.A. | 4.8E-05 | N.A. | 1< | N.A. | |
| C_0096 | 4-Aminosalicylic acid | 4-Aminosalicylic acid | 3.4E-05 | N.A. | N.A. | N.A. | N.A. | N.A. | 1< | N.A. | |
| C_0082 | 4-Methyl-5-thiazoleethanol | 4-Methyl-5-thiazoleethanol | 6.2E-05 | 5.8E-05 | 1.8E-05 | N.A. | 4.4E-05 | N.A. | 3.4 | N.A. | |
| A_0038 | 4-Methylthio-2-oxobutyric acid | KMTB | 8.6E-05 | N.A. | 1.1E-04 | N.A. | N.A. | N.A. | 0.8 | N.A. | |

| | | | | | | | | | | | |
|---|---|---|---|---|---|---|---|---|---|---|---|
| A_0031 | 6-Hydroxynicotinic acid | 6-Hydroxynicotinic acid | 3.0E-05 | N.A. | N.A. | N.A. | N.A. | N.A. | 1< | N.A. | |
| C_0019 | Acetoacetamide | Acetoacetamide | 2.5E-04 | N.A. | N.A. | N.A. | N.A. | N.A. | 1< | N.A. | |
| C_0177 | Adenosine | Adenosine | 3.4E-05 | 1.3E-05 | N.A. | N.A. | N.A. | N.A. | 1< | N.A. | |
| A_0037 | Adipic acid | Adipic acid | N.A. | N.A. | 4.0E-04 | N.A. | 3.6E-04 | N.A. | <1 | N.A. | |
| C_0100 | Allantoin | Allantoin | 3.0E-04 | N.A. | 2.2E-04 | 1.1E-04 | 3.9E-04 | 2.4E-05 | 1.3 | N.A. | |
| C_0120 | Alliin | Alliin | N.A. | N.A. | 2.5E-05 | 5.9E-06 | 1.9E-04 | N.A. | <1 | N.A. | |
| C_0004 | Aminoacetone | Aminoacetone | 1.4E-03 | N.A. | 3.3E-04 | N.A. | N.A. | N.A. | 4.3 | N.A. | |
| A_0126 | AMP | AMP | 4.5E-05 | N.A. | N.A. | N.A. | N.A. | N.A. | 1< | N.A. | |
| C_0043 | Anserine_divalent | Anserine | 1.2E-04 | N.A. | 1.8E-04 | N.A. | N.A. | N.A. | 0.7 | N.A. | |

| | | | | | | | | | | | |
|---|---|---|---|---|---|---|---|---|---|---|---|
| C_0189 | Argininosuccinic acid | ArgSuccinate | 1.7E-05 | 3.9E-06 | N.A. | N.A. | 4.5E-05 | N.A. | 1< | N.A. | |
| A_0107 | Ascorbate 2-sulfate | Ascorbate 2-sulfate | 6.4E-05 | N.A. | N.A. | N.A. | N.A. | N.A. | 1< | N.A. | |
| A_0069 | Ascorbic acid | Ascorbic acid | 1.5E-04 | N.A. | N.A. | N.A. | N.A. | N.A. | 1< | N.A. | |
| C_0188 | Atropine | Atropine | N.A. | N.A. | N.A. | N.A. | 1.7E-04 | N.A. | N.A. | N.A. | |
| C_0036 | Betaine | Betaine | N.A. | N.A. | N.A. | N.A. | 8.5E-04 | N.A. | N.A. | N.A. | |
| C_0137 | Caffeine | Caffeine | 1.5E-04 | N.A. | N.A. | N.A. | N.A. | N.A. | 1< | N.A. | |
| C_0060 | *cis*-4-Hydroxyproline | cis-Hydroxyproline | N.A. | N.A. | 8.9E-05 | 4.3E-05 | 7.8E-05 | N.A. | <1 | N.A. | |
| A_0086 | Citric acid | Citric acid | 4.4E-04 | 3.1E-04 | 7.2E-04 | N.A. | N.A. | N.A. | 0.6 | N.A. | |
| A_0120 | CMP | CMP | 4.3E-05 | N.A. | 2.8E-05 | N.A. | N.A. | N.A. | 1.5 | N.A. | |

| | | | | | | | | | | | |
|---|---|---|---|---|---|---|---|---|---|---|---|
| C_0034 | Creatinine | Creatinine | 1.2E-03 | 8.6E-04 | 9.5E-03 | N.A. | 2.6E-04 | N.A. | 0.13 | N.A. | |
| C_0044 | Cys | Cys | 4.3E-05 | 2.0E-06 | 3.1E-05 | N.A. | N.A. | N.A. | 1.4 | N.A. | |
| C_0031 | Cytosine | Cytosine | 5.0E-05 | 4.4E-05 | N.A. | N.A. | N.A. | N.A. | 1< | N.A. | |
| A_0123 | dAMP | dAMP | 1.6E-05 | 9.5E-07 | N.A. | N.A. | N.A. | N.A. | 1< | N.A. | |
| A_0117 | dCMP | dCMP | 3.1E-05 | 1.5E-05 | 2.4E-05 | N.A. | 3.3E-05 | N.A. | 1.3 | N.A. | |
| A_0061 | Decanoic acid | Decanoic acid | N.A. | N.A. | 4.8E-04 | 4.2E-04 | 1.9E-04 | N.A. | <1 | N.A. | |
| A_0100 | Desthiobiotin | Desthiobiotin | N.A. | N.A. | 1.6E-04 | N.A. | 1.6E-04 | N.A. | <1 | N.A. | |
| A_0129 | Digalacturonic acid | Digalacturonic acid | 7.9E-05 | 4.3E-05 | 9.8E-05 | N.A. | N.A. | N.A. | 0.8 | N.A. | |
| A_0057 | Dihydroxyacetone phosphate | DHAP | 7.8E-05 | 6.4E-06 | 6.5E-05 | N.A. | 2.0E-04 | N.A. | 1.2 | N.A. | |

| | | | | | | | | | | | |
|---|---|---|---|---|---|---|---|---|---|---|---|
| C_0013 | Dimethylaminoethanol | Dimethylaminoethanol | 2.0E-05 | N.A. | N.A. | N.A. | N.A. | N.A. | 1< | N.A. | |
| A_0106 | Dodecanedioic acid | Dodecanedioic acid | 1.3E-04 | 7.7E-05 | N.A. | N.A. | N.A. | N.A. | 1< | N.A. | |
| A_0119 | dTMP | dTMP | 4.9E-05 | 1.2E-05 | 2.2E-05 | N.A. | N.A. | N.A. | 2.3 | N.A. | |
| A_0103 | Ethyl glucuronide | Ethyl glucuronide | 1.1E-04 | N.A. | 1.3E-04 | 6.4E-06 | 1.6E-04 | 5.2E-05 | 0.9 | N.A. | |
| A_0065 | Formiminoglutamic acid | Formiminoglutamic acid | 6.6E-05 | N.A. | 6.4E-05 | N.A. | 1.2E-04 | 2.1E-05 | 1.0 | N.A. | |
| A_0048 | Formylanthranilic acid | Formylanthranilate | 3.9E-05 | N.A. | 1.7E-05 | N.A. | N.A. | N.A. | 2.3 | N.A. | |
| A_0110 | Fructose 6-phosphate | F6P | 2.6E-04 | 1.2E-05 | 2.4E-05 | N.A. | N.A. | N.A. | 11 | N.A. | |
| A_0089 | Galacturonic acid-1 Glucuronic acid-1 | Galacturonic acid Glucuronic acid | 8.8E-04 | N.A. | 1.9E-04 | N.A. | N.A. | N.A. | 4.7 | N.A. | |
| A_0090 | Galacturonic acid-2 Glucuronic acid-2 | Galacturonic acid Glucuronic acid | 1.1E-03 | 1.7E-03 | 5.2E-04 | N.A. | N.A. | N.A. | 2.2 | N.A. | |

| | | | | | | | | | | | | |
|---|---|---|---|---|---|---|---|---|---|---|---|---|
| C_0121 | Gluconolactone | 1,5-Gluconolactone | N.A. | N.A. | N.A. | N.A. | 6.7E-04 | N.A. | N.A. | N.A. | |
| A_0108 | Glucose 1-phosphate | G1P | 6.6E-05 | N.A. | N.A. | N.A. | N.A. | N.A. | 1< | N.A. | |
| A_0109 | Glucose 6-phosphate | G6P | 8.0E-04 | 6.2E-04 | 1.3E-04 | N.A. | 2.4E-04 | N.A. | 6.2 | N.A. | |
| A_0059 | Glycerol 2-phosphate | Glycerol 2-phosphate | N.A. | N.A. | 3.3E-05 | N.A. | N.A. | N.A. | <1 | N.A. | |
| C_0171 | Glycerophosphocholine | GPCho | 3.8E-05 | N.A. | 2.1E-04 | N.A. | 7.8E-05 | N.A. | 0.2 | N.A. | |
| A_0024 | Heptanoic acid | Heptanoic acid | N.A. | N.A. | 6.8E-04 | N.A. | 4.7E-04 | N.A. | <1 | N.A. | |
| C_0032 | Histamine | Histamine | 6.7E-04 | 1.2E-03 | 4.3E-05 | N.A. | 5.2E-05 | N.A. | 15 | N.A. | |
| C_0160 | Homocarnosine | Homocarnosine | 4.2E-05 | N.A. | 6.6E-05 | N.A. | N.A. | N.A. | 0.6 | N.A. | |
| C_0020 | Homoserinelactone | Homoserinelactone | 7.0E-05 | 5.5E-06 | N.A. | N.A. | N.A. | N.A. | 1< | N.A. | |

| | | | | | | | | | | | |
|---|---|---|---|---|---|---|---|---|---|---|---|
| C_0071 | Hydroxyindole | Hydroxyindole | 5.5E-04 | 2.0E-04 | N.A. | N.A. | N.A. | N.A. | 1< | N.A. | |
| C_0099 | Imidazolelactic acid | Imidazolelactic acid | 1.2E-04 | N.A. | 7.3E-04 | 4.8E-04 | 6.1E-04 | N.A. | 0.2 | N.A. | |
| **A_0068** | **Indole-3-acetic acid** | **IAA** | **5.9E-05** | **N.A.** | **1.9E-04** | **2.6E-04** | **4.2E-05** | **N.A.** | **0.3** | **N.A.** | |
| A_0096 | Indole-3-lactic acid<br>5-Methoxyindoleacetic acid | Indolelactic acid<br>5-MIAA | N.A. | N.A. | 2.9E-04 | 1.9E-04 | 1.7E-04 | 1.8E-04 | <1 | N.A. | |
| A_0063 | Isovalerylalanine-2<br>N-Acetylleucine-2 | Isovalerylalanine<br>N-Acetylleucine | 6.6E-05 | N.A. | 6.9E-05 | N.A. | N.A. | N.A. | 1.0 | N.A. | |
| A_0094 | Lauric acid | Lauric acid | 1.1E-04 | N.A. | 2.0E-04 | N.A. | N.A. | N.A. | 0.6 | N.A. | |
| A_0040 | Mevalonic acid | Mevalonic acid | N.A. | N.A. | 1.4E-04 | N.A. | N.A. | N.A. | <1 | N.A. | |
| C_0008 | Morpholine | Morpholine | N.A. | N.A. | 8.4E-05 | N.A. | 1.2E-04 | N.A. | <1 | N.A. | |
| A_0098 | Mucic acid | Mucic acid | 5.4E-05 | N.A. | 1.0E-04 | 4.3E-05 | 3.0E-04 | N.A. | 0.5 | N.A. | |

| | | | | | | | | | | | | |
|---|---|---|---|---|---|---|---|---|---|---|---|---|
| A_0066 | *N*-Acetylasparagine | N-Acetylasparagine | 4.0E-04 | N.A. | N.A. | N.A. | N.A. | N.A. | 1< | N.A. | |
| A_0067 | *N*-Acetylaspartic acid | N-Acetylaspartic acid | 5.2E-04 | 4.0E-04 | 2.0E-04 | N.A. | 1.1E-04 | N.A. | 2.6 | N.A. | |
| A_0079 | *N*-Acetylglutamine | N-Acetylglutamine | 5.1E-05 | 3.5E-05 | N.A. | N.A. | N.A. | N.A. | 1< | N.A. | |
| A_0015 | *N*-Acetylglycine | N-Acetylglycine | 1.5E-04 | N.A. | N.A. | N.A. | N.A. | N.A. | 1< | N.A. | |
| A_0097 | *N*-Acetylphenylalanine | N-Acetylphenylalanine | 3.5E-05 | 2.9E-05 | N.A. | N.A. | 1.8E-05 | N.A. | 1< | N.A. | |
| A_0083 | *N*-Carbamylglutamic acid | N-Carbamylglutamic acid | N.A. | N.A. | 7.5E-05 | N.A. | N.A. | N.A. | <1 | N.A. | |
| A_0032 | *N*-Ethylmaleimide_+$H_2O$ | N-Ethylmaleimide | 4.5E-05 | 2.3E-05 | N.A. | N.A. | N.A. | N.A. | 1< | N.A. | |
| A_0070 | *N*-Formylmethionine | N-Formylmethionine | 1.5E-05 | N.A. | 2.9E-05 | 1.8E-05 | 2.9E-05 | N.A. | 0.5 | N.A. | |
| C_0104 | *N*-Methylglutamic acid | N-Methylglutamic acid | 6.5E-05 | N.A. | 3.8E-04 | N.A. | N.A. | N.A. | 0.2 | N.A. | |

| | | | | | | | | | | | |
|---|---|---|---|---|---|---|---|---|---|---|---|
| C_0072 | *N*-Nitrosodiethanolamine | N-Nitrosodiethanolamine | N.A. | N.A. | 1.3E-04 | N.A. | N.A. | N.A. | <1 | N.A. | |
| C_0186 | $N^{1}$,$N^{12}$-Diacetylspermine | N1,N12-Diacetylspermine | 2.1E-04 | 1.6E-04 | 5.9E-05 | N.A. | N.A. | N.A. | 3.5 | N.A. | |
| C_0164 | $N^{1}$-Acetylspermine | N1-Acetylspermine | 3.2E-05 | 1.3E-05 | 3.0E-05 | N.A. | N.A. | N.A. | 1.0 | N.A. | |
| A_0112 | $N^{2}$-Phenylacetylglutamine | N2-Phenylacetylglutamine | N.A. | N.A. | 7.2E-05 | N.A. | N.A. | N.A. | <1 | N.A. | |
| C_0117 | $N^{5}$-Ethylglutamine | N5-Ethylglutamine | 1.6E-04 | 6.7E-05 | 3.4E-04 | N.A. | N.A. | N.A. | 0.5 | N.A. | |
| C_0047 | Nicotinamide | Nicotinamide | 9.0E-05 | 1.3E-05 | N.A. | N.A. | N.A. | N.A. | 1< | N.A. | |
| C_0196 | NMN | NicRN | 2.4E-04 | 5.1E-05 | N.A. | N.A. | N.A. | N.A. | 1< | N.A. | |
| C_0158 | *N*'-Formylkynurenine | N-Formylkynurenine | 5.1E-05 | N.A. | 2.1E-05 | N.A. | N.A. | N.A. | 2.4 | N.A. | |
| C_0142 | *O*-Acetylcarnitine | ALCAR | N.A. | N.A. | 1.0E-04 | N.A. | 9.0E-05 | N.A. | <1 | N.A. | |

| | | | | | | | | | | | |
|---|---|---|---|---|---|---|---|---|---|---|---|
| C_0091 | *O*-Acetylserine | O-Acetylserine | 6.5E-05 | 3.1E-05 | N.A. | N.A. | N.A. | N.A. | 1< | N.A. | |
| A_0047 | *o*-Coumaric acid<br>*p*-Coumaric acid | o-Coumaric acid<br>p-Coumaric acid | 3.8E-05 | N.A. | 3.2E-05 | N.A. | N.A. | N.A. | 1.2 | N.A. | |
| A_0033 | Octanoic acid | Octanoic acid | N.A. | N.A. | 6.7E-04 | 1.0E-03 | N.A. | N.A. | <1 | N.A. | |
| C_0187 | Ophthalmic acid | Ophthalmic acid | N.A. | N.A. | 3.5E-05 | N.A. | N.A. | N.A. | <1 | N.A. | |
| A_0044 | Oxypurinol | Oxypurinol | 8.2E-05 | 6.4E-05 | 4.6E-05 | N.A. | 5.9E-05 | N.A. | 1.8 | N.A. | |
| C_0078 | *p*-Aminobenzoic acid | p-Aminobenzoic acid | 4.3E-05 | N.A. | 6.6E-05 | 6.7E-05 | 4.8E-05 | 5.0E-06 | 0.6 | N.A. | |
| C_0030 | *p*-Aminophenol<br>*m*-Aminophenol<br>*o*-Aminophenol | p-Aminophenol<br>m-Aminophenol<br>2-Aminophenol | 9.7E-03 | N.A. | N.A. | N.A. | N.A. | N.A. | 1< | N.A. | |
| A_0071 | *p*-Hydroxyphenylpyruvic acid | HPP | N.A. | N.A. | 7.8E-05 | N.A. | N.A. | N.A. | <1 | N.A. | |
| A_0046 | Phenylpyruvic acid | Phenylpyruvate | 6.4E-05 | N.A. | 1.1E-04 | N.A. | N.A. | N.A. | 0.6 | N.A. | |

| | | | | | | | | | | | |
|---|---|---|---|---|---|---|---|---|---|---|---|
| C_0127 | Phosphorylcholine | Phosphorylcholine | 1.1E-04 | 4.2E-05 | 9.8E-05 | N.A. | N.A. | N.A. | 1.2 | N.A. | |
| C_0049 | Pyrazinamide | Pyrazinamide | 6.6E-05 | N.A. | N.A. | N.A. | N.A. | N.A. | 1< | N.A. | |
| C_0112 | Pyridoxine | Pyridoxine | 5.6E-05 | N.A. | N.A. | N.A. | N.A. | N.A. | 1< | N.A. | |
| A_0004 | Pyruvic acid | Pyruvic acid | 7.3E-04 | N.A. | N.A. | N.A. | N.A. | N.A. | 1< | N.A. | |
| C_0198 | Riboflavin | Riboflavin | 8.3E-05 | N.A. | N.A. | N.A. | N.A. | N.A. | 1< | N.A. | |
| A_0105 | Ribose 5-phosphate | R5P | 6.9E-05 | 4.5E-05 | 3.8E-05 | N.A. | 4.7E-05 | N.A. | 1.8 | N.A. | |
| A_0104 | Ribulose 5-phosphate | Ru5P | 1.2E-04 | 4.5E-05 | 8.7E-05 | N.A. | 5.3E-04 | N.A. | 1.3 | N.A. | |
| C_0122 | *S*-Carboxymethylcysteine | S-Carboxymethylcysteine | 2.9E-04 | N.A. | N.A. | N.A. | N.A. | N.A. | 1< | N.A. | |
| C_0010 | Sarcosine | Sarcosine | 2.3E-04 | 7.0E-05 | 9.9E-05 | N.A. | 1.9E-04 | N.A. | 2.3 | N.A. | |

| | | | | | | | | | | | | |
|---|---|---|---|---|---|---|---|---|---|---|---|---|
| A_0115 | Sedoheptulose 7-phosphate | S7P | 1.3E-04 | 1.8E-05 | N.A. | N.A. | N.A. | N.A. | 1< | N.A. | |
| A_0137 | Sucrose 6'-phosphate | Sucrose 6'-phosphate | 2.6E-05 | N.A. | N.A. | N.A. | N.A. | N.A. | 1< | N.A. | |
| A_0111 | Sulfotyrosine | Sulfotyrosine | 1.3E-04 | N.A. | 1.7E-04 | N.A. | N.A. | N.A. | 0.8 | N.A. | |
| A_0042 | Tartaric acid | Tartaric acid | 1.5E-04 | N.A. | N.A. | N.A. | N.A. | N.A. | 1< | N.A. | |
| C_0050 | Taurine | Taurine | 4.5E-04 | 3.8E-04 | 9.8E-04 | N.A. | N.A. | N.A. | 0.5 | N.A. | |
| C_0197 | Thiamine phosphate | TMP | 5.0E-05 | 5.4E-05 | 1.8E-05 | N.A. | N.A. | N.A. | 2.7 | N.A. | |
| A_0041 | Thiodiglycolic acid | Thiodiglycolic acid | 1.1E-04 | 6.4E-05 | N.A. | N.A. | N.A. | N.A. | 1< | N.A. | |
| C_0156 | Thr-Asp | Thr-Asp | 6.2E-05 | 4.7E-06 | 1.1E-04 | N.A. | 5.7E-05 | N.A. | 0.6 | N.A. | |
| C_0058 | *trans*-Glutaconic acid | trans-Glutaconic acid | 2.0E-04 | N.A. | N.A. | N.A. | N.A. | N.A. | 1< | N.A. | |

| | | | | | | | | | | | | |
|---|---|---|---|---|---|---|---|---|---|---|---|---|
| A_0101 | Tridecanoic acid | Tridecanoic acid | N.A. | N.A. | 3.0E-05 | N.A. | N.A. | N.A. | <1 | N.A. | |
| C_0076 | Trigonelline | Trigonelline | 1.2E-04 | N.A. | 4.5E-05 | 3.2E-05 | N.A. | N.A. | 2.6 | N.A. | |
| C_0190 | Trimethoprim | Trimethoprim | N.A. | N.A. | N.A. | N.A. | 1.3E-04 | N.A. | N.A. | N.A. | |
| **C_0102** | **Tryptamine** | **Tryptamine** | **6.2E-05** | **3.9E-05** | **5.7E-05** | **N.A.** | **5.2E-05** | **N.A.** | **1.1** | **N.A.** | |
| C_0138 | Tyrosine methyl ester | Tyrosine methyl ester | 1.1E-04 | 4.4E-05 | N.A. | N.A. | N.A. | N.A. | 1< | N.A. | |
| A_0121 | UMP | UMP | 4.1E-05 | N.A. | N.A. | N.A. | N.A. | N.A. | 1< | N.A. | |
| C_0195 | Val-Pro-Pro | Val-Pro-Pro | 2.1E-05 | N.A. | 1.5E-04 | N.A. | 1.5E-04 | N.A. | 0.14 | N.A. | |
| A_0055 | Vanillic acid | Vanillic acid | 2.2E-04 | 2.7E-04 | 1.7E-05 | N.A. | 2.3E-05 | 3.5E-06 | 13 | N.A. | |
| A_0021 | XA0003 | XA0003 | 3.6E-05 | N.A. | N.A. | N.A. | N.A. | N.A. | 1< | N.A. | |

| | | | | | | | | | | | |
|---|---|---|---|---|---|---|---|---|---|---|---|
| A_0034 | XA0004 | XA0004 | 1.2E-03 | N.A. | 2.3E-05 | N.A. | N.A. | N.A. | 54 | N.A. | |
| A_0052 | XA0012 | XA0012 | 1.9E-05 | 7.7E-06 | 5.8E-05 | N.A. | N.A. | N.A. | 0.3 | N.A. | |
| A_0088 | XA0019 | XA0019 | N.A. | N.A. | 3.7E-05 | N.A. | N.A. | N.A. | <1 | N.A. | |
| A_0114 | Xanthosine | Xanthosine | 5.2E-05 | N.A. | N.A. | N.A. | N.A. | N.A. | 1< | N.A. | |
| C_0053 | XC0016 | XC0016 | 8.6E-05 | N.A. | 8.5E-05 | N.A. | N.A. | N.A. | 1.0 | N.A. | |
| C_0057 | XC0017 | XC0017 | 5.5E-05 | N.A. | 4.1E-05 | N.A. | N.A. | N.A. | 1.4 | N.A. | |
| C_0115 | XC0040 | XC0040 | N.A. | N.A. | 2.5E-05 | N.A. | N.A. | N.A. | <1 | N.A. | |
| C_0149 | XC0065 | XC0065 | 5.2E-05 | N.A. | N.A. | N.A. | N.A. | N.A. | 1< | N.A. | |
| C_0114 | XC0147 | XC0147 | N.A. | N.A. | 3.4E-05 | N.A. | N.A. | N.A. | <1 | N.A. | |

| | | | | | | | | | | | |
|---|---|---|---|---|---|---|---|---|---|---|---|
| C_0125 | XC0148 | XC0148 | 7.6E-05 | N.A. | N.A. | N.A. | N.A. | N.A. | 1< | N.A. | |
| C_0147 | β-Ala-Lys | b-Ala-Lys | N.A. | N.A. | 4.4E-05 | N.A. | 9.3E-05 | N.A. | <1 | N.A. | |
| A_0017 | β-Hydroxyisovaleric acid | b-Hydroxyisovaleric acid | 9.4E-05 | N.A. | 1.3E-04 | N.A. | N.A. | N.A. | 0.7 | N.A. | |
| C_0148 | γ-Glu-Ala | g-Glu-Ala | 1.6E-04 | 1.9E-04 | 2.9E-05 | N.A. | N.A. | N.A. | 5.4 | N.A. | |
| C_0192 | γ-Glu-Citrulline | g-Glu-Citrulline | 2.9E-04 | N.A. | 2.5E-05 | N.A. | N.A. | N.A. | 11 | N.A. | |
| C_0179 | γ-Glu-Gln | g-Glu-Gln | 2.1E-04 | N.A. | N.A. | N.A. | N.A. | N.A. | 1< | N.A. | |
| C_0143 | γ-Glu-Gly | g-Glu-Gly | 3.0E-04 | N.A. | N.A. | N.A. | N.A. | N.A. | 1< | N.A. | |
| C_0174 | γ-Glu-Ornitine | g-Glu-Ornitine | 6.7E-05 | N.A. | 1.1E-04 | N.A. | 8.8E-05 | N.A. | 0.6 | N.A. | |
| C_0155 | γ-Glu-Ser | g-Glu-Ser | 1.1E-04 | N.A. | N.A. | N.A. | N.A. | N.A. | 1< | N.A. | |

| | | | | | | | | | | | |
|---|---|---|---|---|---|---|---|---|---|---|---|
| C_0167 | γ-Glu-Thr | g-Glu-Thr | 7.5E-05 | N.A. | N.A. | N.A. | N.A. | N.A. | 1< | N.A. | |
| C_0165 | γ-Glu-Val | g-Glu-Val | 8.3E-05 | N.A. | 4.3E-04 | 4.9E-04 | 4.1E-04 | 4.6E-04 | 0.2 | N.A. | |

The ID consists of the initial letter of the measurement mode and a serial number, where C indicates cationic mode and A indicates anionic mode.

†Candidate compound names obtained by matching with the HMT database.

¶The ratio of the detected mean values between the two groups was calculated using the latter as the denominator.

‖ The p values and their ranges for Welch's t-test are shown. (*<0.05)

**Supplementary Table 6. Primer sequences for real time-PCR**

| Gene | | Primer sequence |
|---|---|---|
| *ACTB* | Forward | 5'-CATGTACGTTGCTATCCAGGC-3' |
| | Reverse | 5'-CTCCTTAATGTCACGCACGAT-3' |
| *IL1B* | Forward | 5'-GAGCAACAAGTGGTGTTCTCC-3' |
| | Reverse | 5'-AACACGCAGGACAGGTACAG-3' |
| *IFNG* | Forward | 5'-TTCAGCTCTGCATCGTTTTGG-3' |
| | Reverse | 5'-CACTCTTTTGGATGCTCTGGTC-3' |
| *IL6* | Forward | 5'-CAGTTCCTGCAGAAAAAGGCAA-3' |
| | Reverse | 5'-AGCTGCGCAGAATGAGATGA-3' |
| *TNFA* | Forward | 5'-ATGTTGTAGCAAACCCTCAAGC-3' |
| | Reverse | 5'-TGATGGCAGAGAGGAGGTTG-3' |

| | | |
|---|---|---|
| *ACE2* | Forward | 5'-GTCCCGGAGCCGTATCAATG-3' |
| | Reverse | 5'-GGAGGCATAAGGATTTTCTCCAC-3' |
| *AHR* | Forward | 5'-TCAACAGCAACAGTCCTTGG-3' |
| | Reverse | 5'-CCCATAGGGTAGTCCAGCTCT-3' |
| *CYP1A1* | Forward | 5'-GGTCAAGGAGCACTACAAAACC-3' |
| | Reverse | 5'-TGGACATTGGCGTTCTCAT-3' |
| *CARD9* | Forward | 5'-GCGCCTCAAAGAGAGTTTTG-3' |
| | Reverse | 5'-TCAGTGTCGGTGTTGTCGC-3' |
| *IL22* | Forward | 5'-CCCATCAGCTCCCACTGC-3' |
| | Reverse | 5'-GGCACCACCTCCTGCATATA-3' |
| *IL22BP* | Forward | 5'-GGAACACTGGTTGCCTGAAC-3' |
| | Reverse | 5'-ACACTGCTGTTGCCAGTAAGT-3' |

## 4. References to the Supplemental Material


1. Ariyoshi T, Hagihara M, Tomono S, Eguchi S, Minemura A, Miura D, et al. Clostridium butyricum MIYAIRI 588 Modifies Bacterial Composition under Antibiotic-Induced Dysbiosis for the Activation of Interactions via Lipid Metabolism between the Gut Microbiome and the Host. *Biomedicines.* (2021) 9:1065. doi: 10.3390/biomedicines9081065

2. Bokulich NA, Kaehler BD, Rideout JR, Dillon M, Bolyen E, Knight R, et al. Optimizing taxonomic classification of marker-gene amplicon sequences with QIIME 2's q2-feature-classifier plugin. *Microbiome.* (2018) 6:90. doi: 10.1186/s40168-018-0470-z

3. Hall M, Beiko RG. 16S rRNA Gene Analysis with QIIME2. *Methods Mol Biol.* (2018) 1849:113–29. doi: 10.1007/978-1-4939-8728-3_8

4. Segata N, Izard J, Waldron L, Gevers D, Miropolsky L, Garrett WS, et al. Metagenomic biomarker discovery and explanation. *Genome Biol.* (2011) 12:R60. doi: 10.1186/gb-2011-12-6-r60

5. Ohashi Y, Hirayama A, Ishikawa T, Nakamura S, Shimizu K, Ueno Y, et al. Depiction of metabolome changes in histidine-starved Escherichia coli by CE-TOFMS. *Mol Biosyst.* (2008) 4:135–47. doi: 10.1039/b714176a

6. Ooga T, Sato H, Nagashima A, Sasaki K, Tomita M, Soga T, et al. Metabolomic anatomy of an animal model revealing homeostatic imbalances in dyslipidaemia. *Mol Biosyst.* (2011) 7:1217–23. doi: 10.1039/c0mb00141d

7. Sugimoto M, Wong DT, Hirayama A, Soga T, Tomita M. Capillary electrophoresis mass spectrometry-based saliva metabolomics identified oral, breast and pancreatic cancer-specific profiles. *Metabolomics.* (2010) 6:78–95. doi: 10.1007/s11306-009-0178-y

8. Yamamoto H, Fujimori T, Sato H, Ishikawa G, Kami K, Ohashi Y. Statistical hypothesis testing of factor loading in principal component analysis and its application to metabolite set enrichment analysis. *BMC Bioinformatics.* (2014) 15:51. doi: 10.1186/1471-2105-15-51
